# Citrobacter amalonaticus Inhibits the Growth of Citrobacter rodentium in the Gut Lumen

**DOI:** 10.1128/mBio.02410-21

**Published:** 2021-10-05

**Authors:** Caroline Mullineaux-Sanders, Danielle Carson, Eve G. D. Hopkins, Izabela Glegola-Madejska, Alejandra Escobar-Zepeda, Hilary P. Browne, Trevor D. Lawley, Gad Frankel

**Affiliations:** a Centre for Molecular Microbiology and Infection, Department of Life Sciences, Imperial College, London, United Kingdom; b Microbiome Informatics Team, EMBL-EBI, Hinxton, United Kingdom; c Host-Microbiota Interactions Lab, Wellcome Trust Sanger Institute, Hinxton, United Kingdom; GSK Vaccines; GSK Vaccines

**Keywords:** *Citrobacter*, gastrointestinal infection, colonization resistance

## Abstract

The gut microbiota plays a crucial role in susceptibility to enteric pathogens, including Citrobacter rodentium, a model extracellular mouse pathogen that colonizes the colonic mucosa. C. rodentium infection outcomes vary between mouse strains, with C57BL/6 and C3H/HeN mice clearing and succumbing to the infection, respectively. Kanamycin (Kan) treatment at the peak of C57BL/6 mouse infection with Kan-resistant C. rodentium resulted in relocalization of the pathogen from the colonic mucosa and cecum to solely the cecal luminal contents; cessation of the Kan treatment resulted in rapid clearance of the pathogen. We now show that in C3H/HeN mice, following Kan-induced displacement of C. rodentium to the cecum, the pathogen stably colonizes the cecal lumens of 65% of the mice in the absence of continued antibiotic treatment, a phenomenon that we term antibiotic-induced bacterial commensalization (AIBC). AIBC C. rodentium was well tolerated by the host, which showed few signs of inflammation; passaged AIBC C. rodentium robustly infected naive C3H/HeN mice, suggesting that the AIBC state is transient and did not select for genetically avirulent C. rodentium mutants. Following withdrawal of antibiotic treatment, 35% of C3H/HeN mice were able to prevent C. rodentium commensalization in the gut lumen. These mice presented a bloom of a commensal species, Citrobacter amalonaticus, which inhibited the growth of C. rodentium *in vitro* in a contact-dependent manner and the luminal growth of AIBC C. rodentium *in vivo*. Overall, our data suggest that commensal species can confer colonization resistance to closely related pathogenic species.

## INTRODUCTION

The mammalian gut is colonized by up to 10^14^ commensal bacteria, which provide benefits to the host, including *de novo* synthesis of vitamins and metabolism of dietary fibers (1). The microbiota also provides colonization resistance against invading pathogens indirectly by training the host immune system and modulating gut physiology, as well as by competing with or directly killing or arresting the growth of pathogens (2, 3). Successful enteric pathogens are able to overcome the colonization resistance barrier and in many cases exploit the microbiota, for example, by using microbiota-derived metabolites as cues to fine-tune virulence gene expression to the correct niche (2). For example, enterohemorrhagic Escherichia coli (EHEC) uses microbiota-derived succinate in the colon to regulate virulence (4). The result is a complex web of interactions between the pathogen and the microbiota, which influences the disease course in a way that may ultimately be beneficial or detrimental to the host.

Enteropathogenic E. coli (EPEC), EHEC, and Citrobacter rodentium are extracellular enteric pathogens which colonize the intestinal mucosa while forming attaching and effacing (A/E) lesions (5). These are characterized by intimate attachment of the bacteria to intestinal epithelial cells (IECs) and effacement of the brush border microvilli (6, 7). The key virulence factors of A/E pathogens, encoded on the locus of enterocyte effacement (LEE) pathogenicity island, are a type 3 section system (T3SS), chaperones, the adhesion intimin, and effector proteins, including Tir (8). Intimin-Tir interactions mediate the intimate bacterial attachment and A/E lesion formation (9, 10). Expression of the LEE genes is regulated by the master regulator Ler (11), which itself is regulated by the LEE-encoded positive and negative regulators GrlA and GrlR, respectively (12), and multiple environmental cues (4, 13). Deletion of *grlR* leads to constitutive expression of Ler and the LEE genes (14).

C. rodentium is a natural murine pathogen which colonizes the colonic mucosa in the presence of the endogenous microbiota. Infection results in the activation of inflammatory and tissue damage repair responses, manifested as diarrhea and colonic crypt hyperplasia (CCH) (5). Susceptibility to C. rodentium infection varies between mouse strains. C57BL/6 mice develop a self-limiting infection and mild disease; C. rodentium colonization peaks at 6 to 8 days postinfection (DPI), during which time C. rodentium adheres to the distal colonic and cecal IECs. The pathogen is subsequently cleared from the mucosa in an IgG-dependent manner at 12 to 14 DPI and then from the lumen via outcompetition by commensals by 21 DPI (15–17). Conversely, C3H-derivative mice are lethally susceptible and typically succumb to diarrheal disease from around 12 DPI (18); mortality in C3H/HeJ mice can be reversed by rehydration therapy (19). Genetic and microbiota variation contributes to differences in disease susceptibility between mouse strains (20), and transplantation of the gut microbiota from Swiss NIH or C57BL/6 mice to C3H/HeJ mice can delay or reverse the mortality phenotype, respectively (21, 22).

We previously demonstrated that treatment with kanamycin (Kan) at the peak of C57BL/6 mouse infection with the bioluminescent and Kan-resistant C. rodentium strain ICC180 resulted in a relocalization of the pathogen from the colonic mucosa and cecum to solely the cecal luminal contents, suggesting that Kan-sensitive commensals may be required to sustain C. rodentium infection at the colonic mucosa. C. rodentium persisted under conditions of continued antibiotic treatment, a phenomenon that we termed antibiotic-induced bacterial persistence (AIBP) (14). Withdrawal of the Kan treatment resulted in rapid clearance of the pathogen (14), presumably as a result of the recovery of the colonization resistance capability of the gut microbiota. Here, we investigated the effect of Kan treatment during C. rodentium infection of C3H/HeN (C3H) mice. As with C57BL/6 mice, we found that Kan treatment at the peak of infection causes displacement of the pathogen from the colonic mucosa and cecum to the cecal lumen. However, unlike in C57BL/6 mice, following the withdrawal of Kan treatment, C. rodentium was not cleared from 65% of C3H mice but rather continued to persistently colonize the cecal lumen, a phenomenon that we term antibiotic-induced bacterial commensalization (AIBC). Although commensalization did not select for genetically avirulent C. rodentium mutants, AIBC C. rodentium did not induce acute inflammation in the host. Intriguingly, following the withdrawal of antibiotic treatment, 35% of C3H mice excluded C. rodentium from the gut lumen, and we identified a commensal bacterium, Citrobacter amalonaticus, which specifically bloomed in these mice. *C. amalonaticus* inhibited the growth of C. rodentium in a contact-dependent manner *in vitro* and was sufficient to confer colonization resistance against the growth of C. rodentium in the gut lumen *in vivo*.

## RESULTS

### Kan treatment of infected C3H mice induces C. rodentium commensalization.

**FIG 1:**
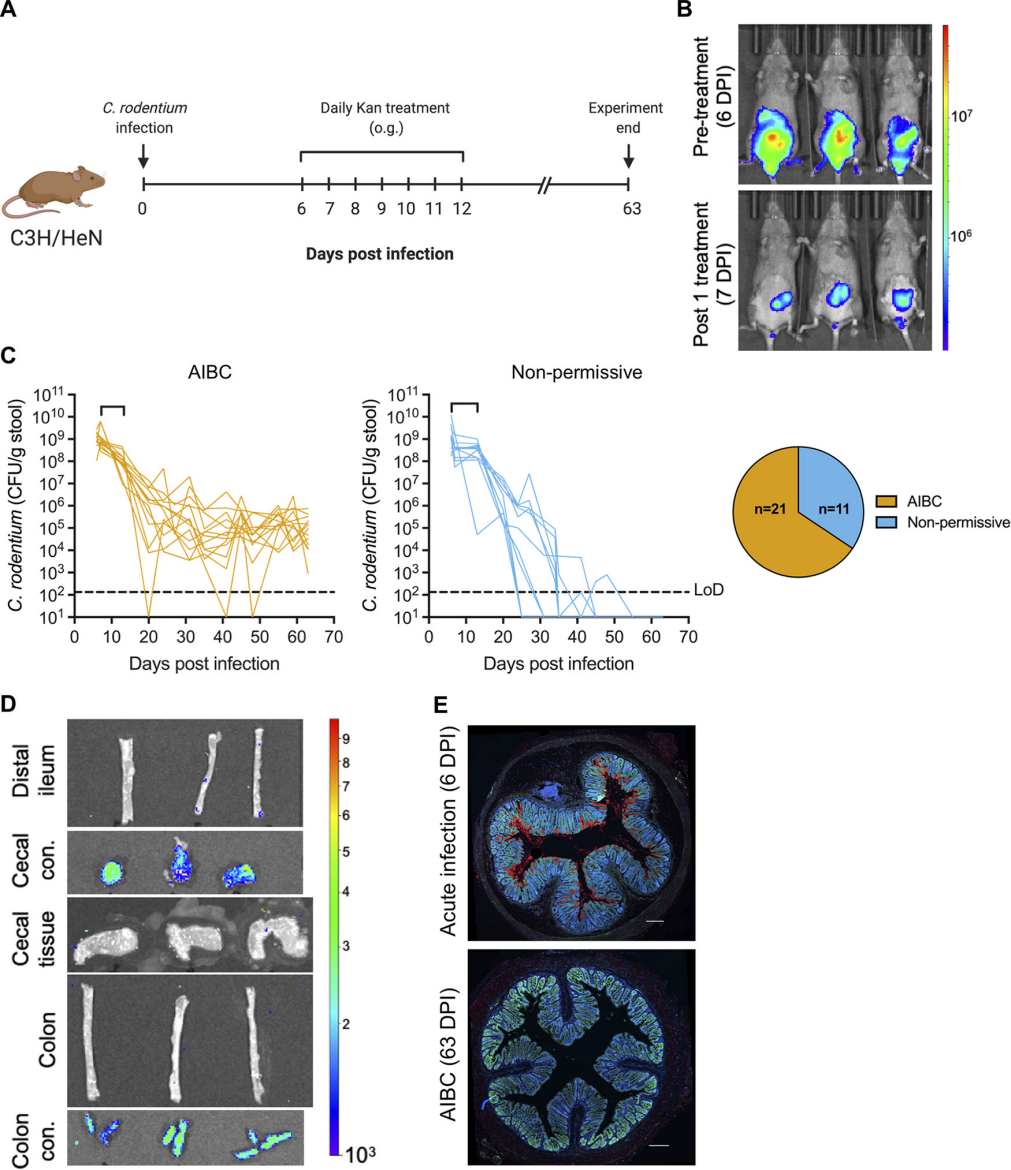
Kan treatment of ICC180-infected C3H mice induces AIBC (A) Schematic of the experimental timeline. o.g., oral gavage. (B) Representative *in vivo* BLIs of mice at 6 DPI prior to Kan treatment (top) and at 7 DPI 24 h after the first Kan treatment (bottom). (C) C. rodentium (strain ICC180) colonization of mice designated AIBC (left) or nonpermissive (middle). Brackets on the line graphs denote the Kan treatment period. Each line represents an individual mouse. Only AIBC mice monitored until 63 DPI are shown (24 mice from five biological repeats). (Right, pie chart) The ratio of AIBC to nonpermissive designations of all mice used in this study (32 mice from six biological repeats). LoD, limit of detection. (D) *Ex vivo* BLIs of organs from AIBC mice at 63 DPI. Images are representative of 4 mice from one biological repeat. con., contents. In panels B and D, color scale bar indicates radiance (photons per second per square centimeter per surface radiance). (E) Immunofluorescence staining of the distal colons of mice acutely infected with C. rodentium (6 DPI with strain ICC169) or mice harboring AIBC C. rodentium at 63 DPI (AIBC), demonstrating no staining of C. rodentium on the colonic mucosa in the AIBC group. C. rodentium, red; DNA, blue; E-cadherin, green. Images are representative of 8 mice from three biological repeats (AIBC) or 5 mice from one biological repeat (acute infection). Scale bar = 200 μm.

We investigated the effect of Kan treatment during infection of C3H mice with the Kan-resistant and bioluminescent C. rodentium strain ICC180. Mice infected with ICC180 were given 7 daily oral Kan treatments from 6 DPI (Fig. 1A). Consistently with previous findings for C57BL/6 mice (14), a single Kan treatment of C3H mice at 6 DPI was sufficient to displace the majority of C. rodentium from the colon and cecum to solely the cecum (Fig. 1B), from where C. rodentium was shed at approximately 10^8^ CFU/g of feces (GoF) for the duration of the treatment period (Fig. 1C). Seventy percent of mice survived until the end of the daily antibiotic treatment (see Fig. S1A in the supplemental material).

10.1128/mBio.02410-21.1 FIG S1 (A) Survival curve of C3H mice infected with ICC180 or ICC180 Δ*grlR* and given daily treatment with Kan from 6 to 12 DPI inclusive. The treatment period is denoted by dotted lines. Data are from 49 mice from 6 biological repeats (ICC180) or 15 mice from three biological repeats (ICC180 Δ*grlR*). *, *P* < 0.05 (as determined by a log rank [Mantel-Cox] test). (B) Immunofluorescence staining of the proximal colon and distal ileum of mice harboring AIBC C. rodentium (shown in Fig. 1C) at 63 DPI, demonstrating no staining of C. rodentium on the mucosa. C. rodentium, red; DNA, blue; E-cadherin, green. Images are representative of 8 mice from three biological repeats. Scale bar = 200 μm. Download FIG S1, TIF file, 1.7 MB. Copyright © 2021 Mullineaux-Sanders et al. 2021 Mullineaux-Sanders et al. https://creativecommons.org/licenses/by/4.0/ This content is distributed under the terms of the Creative Commons Attribution 4.0 International license.

Unlike in C57BL/6 mice (14), withdrawal of Kan treatment did not result in complete pathogen clearance. Instead, 65% of surviving mice continued to persistently shed C. rodentium at around 10^4^ to 10^6^ CFU/GoF up to 9 weeks postinfection, when the experiment was terminated (Fig. 1C), a phenomenon that we term antibiotic-induced bacterial commensalization (AIBC). In contrast, 35% of the surviving mice were nonpermissive to AIBC C. rodentium and cleared the pathogen (Fig. 1C) (see Materials and Methods for a detailed explanation of the AIBC and nonpermissive criteria). *Ex vivo* bioluminescent imaging (BLI) 9 weeks postinfection demonstrated that AIBC C. rodentium resided solely in the cecal and colonic luminal contents (Fig. 1D). Further, while C. rodentium was readily identifiable by immunofluorescence staining on the distal colonic mucosas of acutely infected C3H mice, no C. rodentium was seen on the distal ileal, proximal colonic, or distal colonic mucosas of mice harboring AIBC C. rodentium (Fig. 1E; Fig. S1B). These results demonstrate that following a short Kan treatment course, C. rodentium persistently colonizes solely the gut lumen of the majority of C3H mice in the absence of continued antibiotic treatment. However, a minority of the mice were nonpermissive to this long-term colonization, suggesting that the microbiotas in these mice were able to confer resistance to the luminal growth of C. rodentium.

### The AIBC C. rodentium state is not inflammatory.

**FIG 2:**
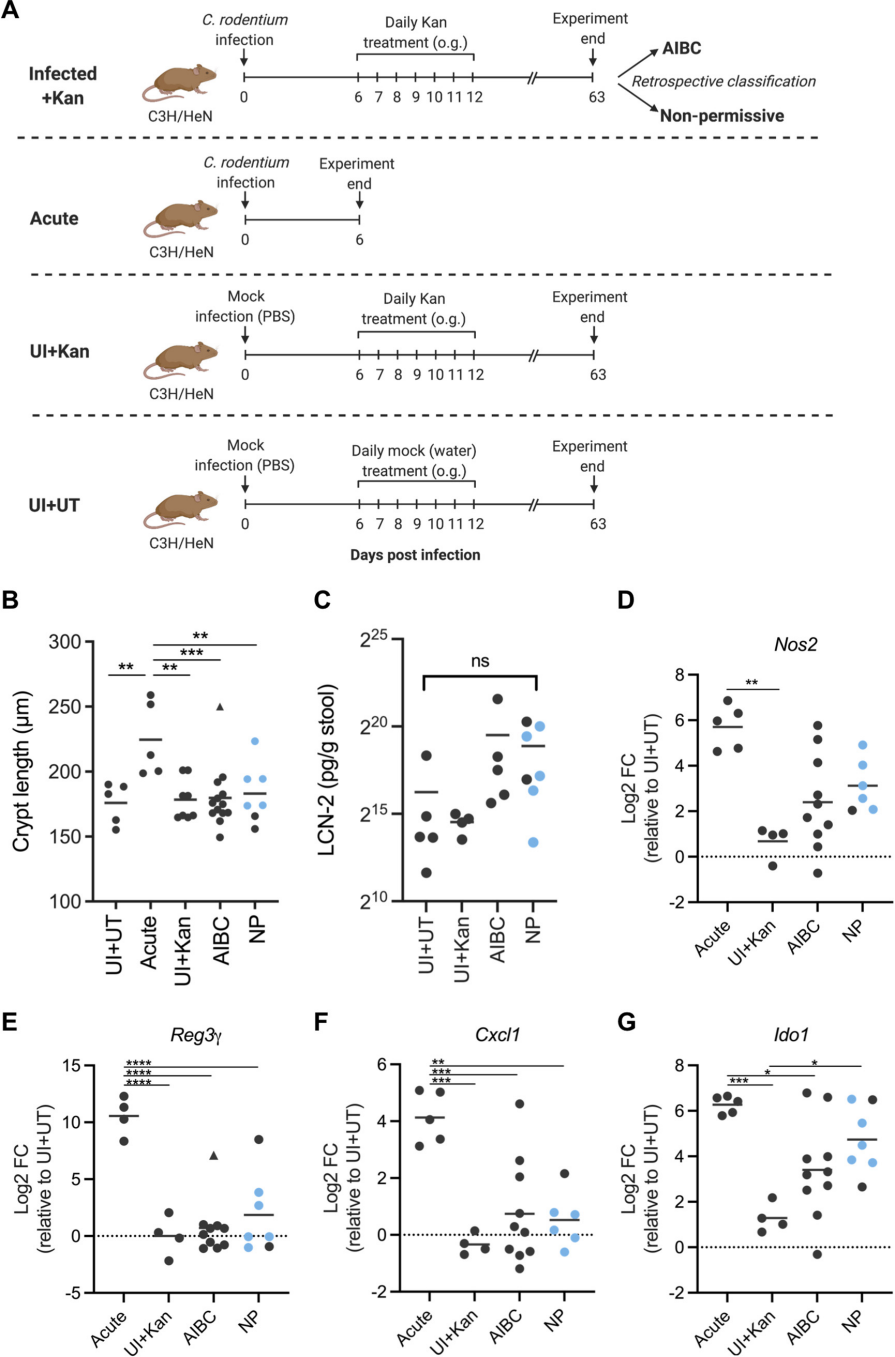
AIBC C. rodentium is not inflammatory. (A) Schematic of the experimental timeline of the AIBC, nonpermissive, Acute, UI+Kan, and UI+UT groups of mice. AIBC and nonpermissive mice are the same mice shown in Fig. 1C. (B) Colonic crypt lengths of mice from the indicated groups. Each point represents the average crypt length of an individual mouse. Data are from one (UI+UT, UI+Kan, Acute), two (nonpermissive), or four (AIBC) biological repeats. (C) Stool LCN-2 concentrations in mice from the indicated groups at 63 DPI. Each point represents an individual mouse. Data are from one (UI+UT, UI+Kan) or two (AIBC, nonpermissive) biological repeats. ns, not significant (*P* < 0.05 for all comparisons, as determined by a one-way ANOVA with Tukey’s posttest for multiple comparisons between all groups). (D to G) qRT-PCR analysis of the indicated mRNA isolated from IECs of mice in the Acute, AIBC, nonpermissive, or UI+Kan group. All are shown relative to the mean of results from the UI+UT group. Each point represents an individual mouse. Data are from one (UI+UT, UI+Kan, and Acute), two (NP), or three (AIBC) biological repeats. (B, E to G) *, *P* < 0.05; **, *P* < 0.01; ***, *P* < 0.001; ****, *P* < 0.0001 (as determined by a one-way ANOVA with Tukey’s posttest for multiple comparisons between all groups). The triangle points in panels B and E indicate data points identified as an outlier and not included in statistical analyses. (D) *Nos2* data did not pass a normality test and were analyzed by a Kruskal-Wallis test with Dunn’s posttest for multiple comparisons between all groups (**, *P* < 0.01). (B to G) Blue points indicate nonpermissive (NP) mice harboring *C. amalonaticus*^C3H^.

Following the withdrawal of Kan treatment, mice with AIBC C. rodentium did not show any visible signs of morbidity and gained weight at a rate similar to that of nonpermissive and uninfected mice (Fig. S2A), suggesting that AIBC C. rodentium are nonpathogenic. We therefore investigated whether mice harboring AIBC C. rodentium (harvested at 63 DPI; AIBC) displayed characteristic markers of colitis associated with acute C. rodentium infection. Nonpermissive mice, acutely infected mice (6 DPI, no treatment; Acute group), mock-infected mice which were mock treated with water from 6 to 12 DPI and harvested at 63 DPI (uninfected plus untreated [UI+UT]), and mock-infected mice which were treated with Kan from 6 to 12 DPI and harvested at 63 DPI (UI+Kan) were used as controls (Fig. 2A).

10.1128/mBio.02410-21.2 FIG S2 (A) Relative weight changes of the UI+UT, UI+Kan, AIBC, and nonpermissive groups of mice. Means ± standard errors of the means (SEM) are shown. We used 13 AIBC mice from four biological repeats (only AIBC mice followed until 63 DPI without commensal inoculation are shown), 11 nonpermissive mice from four biological repeats,  4 UI+Kan mice from one biological repeat, and 5 UI+UT mice from one biological repeat. Dotted lines denote the Kan treatment period. (B) Representative distal colonic sections of mice shown in Fig. 2B stained with hematoxylin and eosin. Scale bar = 200 μm. (C) Stool LCN-2 concentrations in mice in the indicated groups. Each line represents an individual mouse followed over time (63-DPI data are also shown in Fig. 2C). Brackets denote the treatment period. Data are from one (UI+UT, UI+Kan) or two (AIBC, Nonpermissive) biological repeats. Download FIG S2, JPG file, 0.9 MB. Copyright © 2021 Mullineaux-Sanders et al. 2021 Mullineaux-Sanders et al. https://creativecommons.org/licenses/by/4.0/ This content is distributed under the terms of the Creative Commons Attribution 4.0 International license.

While the Acute group presented significantly elongated crypts compared to those of UI+UT mice, the UI+Kan group, nonpermissive group, and all but one mouse of the AIBC group showed no hyperplasia (Fig. 2B; Fig. S2B). Overall, the UI+UT and UI+Kan mice showed no change in the levels of LCN-2 (an inflammatory marker) in stools throughout the 63-day experimental period (Fig. S2C). Conversely, AIBC and nonpermissive mice showed an ∼30-fold increase in LCN-2 in the stool at 6 DPI, prior to treatment, which decreased steeply at the end of the Kan treatment period (Fig. S2C). Following the withdrawal of Kan treatment in AIBC and nonpermissive mice, LCN-2 levels fluctuated but showed no significant difference from levels in UI+UT mice at 63 DPI (Fig. S2C; Fig. 2C).

Acute C. rodentium infection induces global changes in the transcriptome and proteome of IECs (23–25); these changes include an increased expression of the antimicrobial peptide gene *RegIIIγ*, the inducible NO synthase gene *Nos2*, the gamma interferon (IFN-γ)-inducible gene *Ido1*, and the neutrophil chemoattractant gene *Cxcl1*. Quantitative real-time PCR (qRT-PCR) analysis of mRNA from IECs from UI+Kan mice showed a mean log_2_ fold change of close to 0 relative to UI+UT mice for all tested genes, suggesting that daily Kan treatment from 6 to 12 DPI did not influence the expression of these genes at 63 DPI. As expected, Acute group mice had a significantly higher expression of *RegIIIγ*, *Ido1*, *Nos2*, and *Cxcl1* than UI+Kan mice (Fig. 2D to G). Conversely, mice harboring AIBC C. rodentium showed significantly lower levels of expression than Acute group mice of all the tested genes, except *Nos2* (*P* = 0.0513) (Fig. 2D to G), although with a spread of expression levels that was broader than that of UI+Kan mice (Fig. 2D to G), which likely reflects the gradual resolution of inflammation following an acute infection. Nonpermissive mice showed a transcription profile similar to that of AIBC mice for all tested genes (Fig. 2D to G). Taken together, these results suggest that AIBC C. rodentium, which resides in the lumen of the gut, does not induce acute inflammation.

### Expression of the LEE is repressed in the AIBC state.

**FIG 3:**
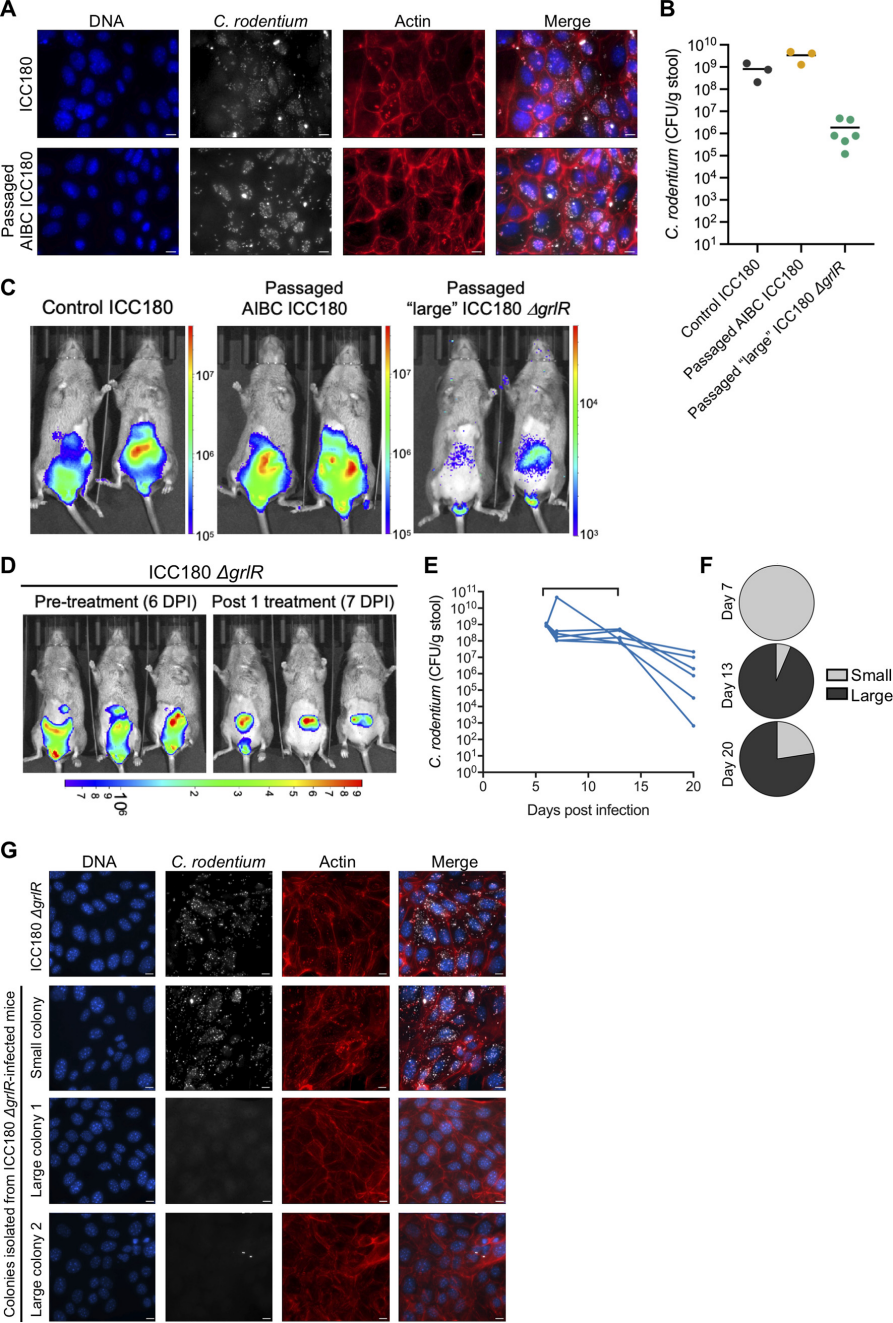
C. rodentium T3SS repression is favored in the AIBC state. (A) CMT-93 cells infected with passaged C. rodentium isolated from mice with AIBC C. rodentium at 63 DPI (mice shown in Fig. 1C). The image is representative of infections with 22 colonies from 6 mice. Scale bar = 10 μm. (B) C. rodentium colonization of mice at 6 DPI with the indicated C. rodentium strain. Each point represents an individual mouse. Data are from one (ICC180 and passaged AIBC ICC180) or two (passaged large ICC180 Δ*grlR*) biological repeats. (C) Representative *in vivo* BLI images of mice shown in panel B at 6 DPI. (D) *In vivo* BLI images of ICC180 Δ*grlR*-infected mice at 6 DPI, prior to Kan treatment (left) and at 7 DPI, 24 h after the first Kan treatment (right). (C, D) Color scale bars indicate radiance (photons per second per square centimeter per surface radiance). (E) C. rodentium colonization of mice infected with ICC180 Δ*grlR* and treated daily with Kan from 6 to 12 DPI inclusive (denoted by the bracket on the graph). Each line represents an individual mouse, and data are from two biological repeats. (F) Percentages of isolated ICC180 Δ*grlR* colonies which were assigned as having a large- or small-colony morphology at the indicated DPI. Pie charts show the average percentages of all mice shown in panel E. (G) CMT-93 cells infected with control ICC180 Δ*grlR* or passaged small or large ICC180 Δ*grlR* colonies isolated from stool samples of mice shown in panel E at 11 to 20 DPI. Immunofluorescence images are representative of 9 colonies from 5 mice (large colonies) and 8 colonies from 4 mice (small colonies). Scale bar = 10 μm.

A study demonstrated that administering C3H mice a short course of dietary iron promoted insulin resistance, increased intestinal glucose levels, and drove the selection of genetically avirulent C. rodentium mutants *in vivo* (26), which persist for long periods in the gut lumen, like AIBC C. rodentium. We therefore investigated whether AIBC C. rodentium acquired mutations which conferred a loss of LEE gene expression by isolating AIBC C. rodentium colonies at 63 DPI and infecting the mouse colonic cell line CMT-93. We found that 100% of passaged AIBC C. rodentium colonies isolated at 9 weeks postinfection (*n* = 22 colonies from 5 mice) robustly formed actin-rich pedestals on CMT-93 cells (Fig. 3A), suggesting that they express the T3SS and effector proteins. Consistently, a singly passaged isolate of AIBC C. rodentium was able to infect naive C3H mice, which developed a normal colonic infection at 6 DPI (Fig. 3B and C), demonstrating that the AIBC state is not due to a genetic loss of virulence gene function.

As AIBC C. rodentium does not acquire genetic mutations preventing LEE gene expression, we next investigated whether C. rodentium constitutively expressing the LEE genes could also commensalize. C3H mice were infected with C. rodentium Δ*grlR*, which constitutively expresses the LEE genes (14). As with wild-type (WT) C. rodentium, a single Kan treatment at 6 DPI caused a relocalization of C. rodentium Δ*grlR* from the colon to the cecum (Fig. 3D). However, significantly fewer Δ*grlR* mutant-infected than WT-infected mice survived, with only 40% surviving to the end of the 7-day treatment period (Fig. S1A).

Deletion of the *grlR* gene and, therefore, constitutive expression of the LEE genes confer a fitness cost when C. rodentium is grown on LB agar, resulting in colonies which are smaller than WT colonies (Fig. S3A). Interestingly, we noticed that during the Kan treatment period, mice shed notable numbers of the WT-sized large colonies of C. rodentium Δ*grlR* in their stool, comprising >75% of the isolated colonies at 13 and 20 DPI (Fig. 3E and F). We therefore hypothesized that C. rodentium Δ*grlR* may have acquired genetic mutations during the treatment period preventing the expression of the LEE genes. To test this, we infected CMT-93 cells with large and small colonies isolated at 11 to 20 DPI. While the small-colony strains robustly formed actin-rich pedestals, the large-colony strains were unable to intimately adhere to, or form pedestals on, CMT-93 cells (Fig. 3G). Further, when used to infect naive C3H mice, a large-colony Δ*grlR* isolate was shed at approximately 10^6^ CFU/GoF from 3 to 34 DPI (in the absence of antibiotic treatment) (Fig. 3B; Fig. S3B), similar to the level of AIBC C. rodentium shedding, and resided in the cecum (Fig. 3C). Taken together, these results suggest that downregulation of the LEE genes is favored in the AIBC state. Importantly however, in WT C. rodentium, the LEE genes are likely downregulated via an active regulatory mechanism, as passaged AIBC C. rodentium was able to colonize to WT C. rodentium levels, showing no selection for genetic avirulence.

10.1128/mBio.02410-21.3 FIG S3 (A) ICC180 and ICC180 Δ*grlR* grown on LB agar, showing the smaller colony size of ICC180Δ*grlR*. (B) Colonization of mice (6 DPI data also shown in Fig. 3B) infected with passaged large ICC180 Δ*grlR* over time (no antibiotic treatment given). Each line represents an individual mouse. Data are from two biological repeats. Download FIG S3, TIFF file, 0.9 MB. Copyright © 2021 Mullineaux-Sanders et al. 2021 Mullineaux-Sanders et al. https://creativecommons.org/licenses/by/4.0/ This content is distributed under the terms of the Creative Commons Attribution 4.0 International license.

### Nonpermissive mice harbor commensal *Citrobacter*.

**FIG 4:**
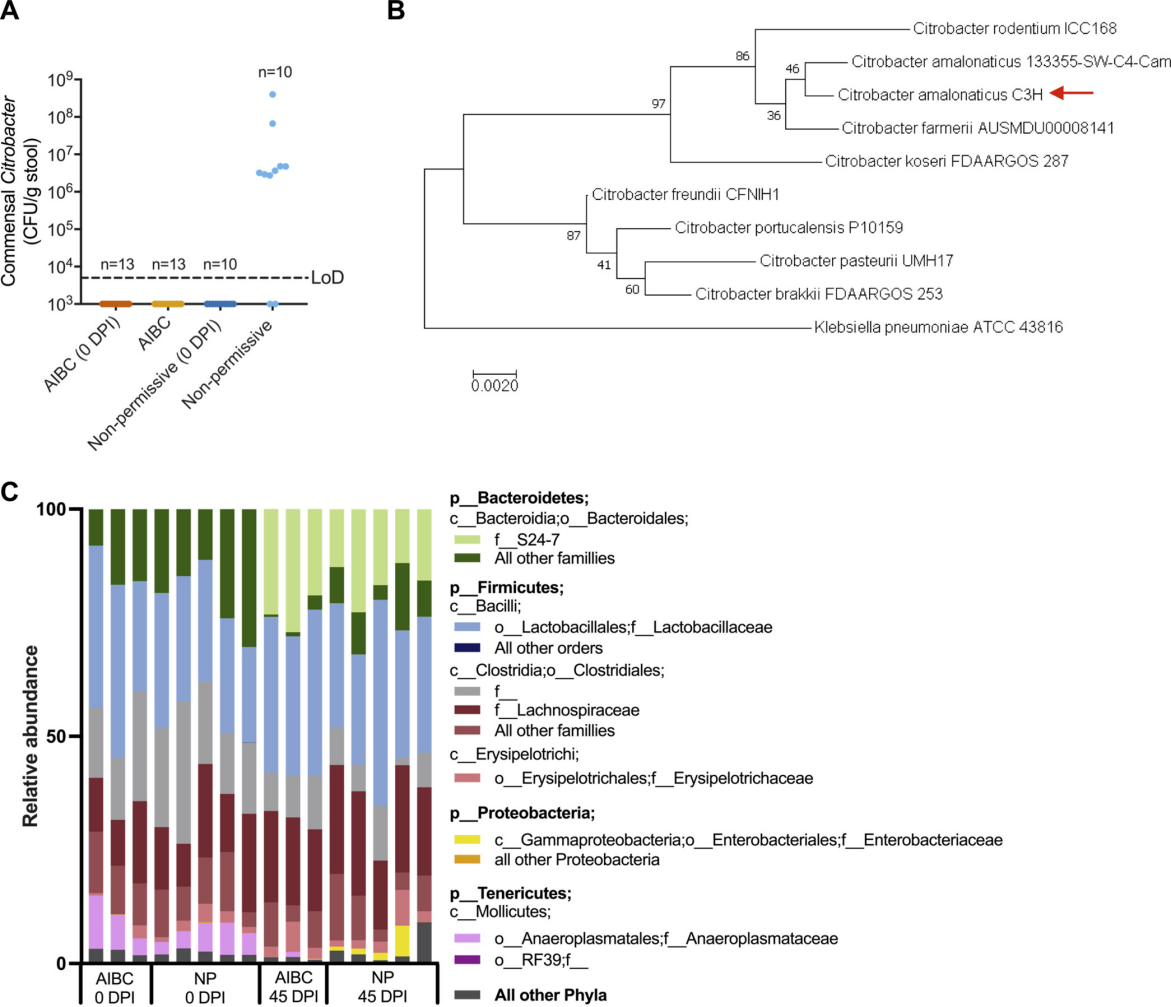
Commensal *Citrobacter* blooms in nonpermissive mice. (A) Non-C. rodentium bacteria assigned to the *Citrobacter* genus in stool samples from AIBC and nonpermissive mice collected from the same mice prior to C. rodentium infection (0 DPI) and at 34 or 41 DPI. Each point represents an individual mouse. Data are from three biological repeats. LoD, limit of detection. (B) The 16S rRNA gene of *C. amalonaticus*^C3H^ was compared to selected *Citrobacter* species using a maximum likelihood model. The percentage of trees in which the associated taxa cluster together in the bootstrap test (1,000 replicates) is shown next to each branch. The tree is drawn to scale, with branch lengths measured in numbers of substitutions per site. Klebsiella pneumoniae is used as an outgroup. The red arrow denotes the *C. amalonaticus*^C3H^ strain isolated in this study. (C) 16S rRNA gene taxonomic analysis of the stool microbiomes from AIBC and nonpermissive (NP) mice at 0 and 45 DPI. Each bar represents a mouse; stool samples from the same mice were analyzed at 0 and 45 DPI. p, phylum; c, class; o, order; f, family.

Despite their identical genetic backgrounds, a minority of C3H mice were nonpermissive to AIBC; i.e., C. rodentium was cleared once the Kan treatment was stopped (Fig. 1C), suggesting that the microbiotas of these mice provided colonization resistance to the luminal growth of C. rodentium. It has been reported that minor facultative anaerobic members of the gut microbiota can vary between genetically similar mouse strains, and this can impact the strains’ susceptibility to Salmonella infection (27). Further, commensal E. coli has been shown to outcompete luminal C. rodentium in C57BL/6 mice by competing for monosaccharides (17). We therefore reasoned that a commensal facultative anaerobe may govern permissibility to AIBC. To test this, we cultured fecal facultative commensal anaerobes from the stools of AIBC and nonpermissive mice (following the clearance of C. rodentium) on nonselective agar under aerobic conditions. Commensals were similarly cultured from stools of the same mice prior to infection. Colonies were grouped by gross morphology and identified by sequencing of the 16S rRNA gene. Eight out of 10 nonpermissive mice were found to contain a commensal isolate of the *Citrobacter* genus, which was not detectable in any tested AIBC mouse or from any mice (AIBC or nonpermissive) prior to C. rodentium infection (Fig. 4A). Nonpermissive mice which harbored commensal *Citrobacter* (blue points in Fig. 2B to G) showed lower levels of inflammatory markers and CCH than mice acutely infected with C. rodentium, suggesting that the presence of commensal *Citrobacter* in the gut did not induce acute inflammation.

We generated a high-quality merged Illumina and Nanopore assembly of the genome of a single colony, which demonstrated that it was most closely related to Citrobacter amalonaticus based on its full-length 16S rRNA gene, and we named this isolate *C. amalonaticus*^C3H^ (Fig. 4B). *C. amalonaticus*^C3H^ was not isolated from any mouse prior to infection (Fig. 4A), suggesting that, under homeostatic conditions, *C. amalonaticus*^C3H^ is present in the gut below the detection level. The Kan MIC for *C. amalonaticus*^C3H^ was ≤8 μg/ml (Fig. S4A), and consistently, the organism bloomed in nonpermissive mice between 13 and 20 DPI (after the withdrawal of Kan treatment) (Fig. S4B), suggesting that a newly created niche following antibiotic depletion of the microbiota may have assisted in its expansion in the gut lumen.

10.1128/mBio.02410-21.4 FIG S4 (A) *C. amalonaticus*^C3H^ grown on LB agar containing the indicated concentration of Kan, demonstrating growth at 4 but not 8 μg/μl. Images are representative of three biological repeats. (B) Commensal *Citrobacter* in the stools of nonpermissive mice over time. Each line represents an individual mouse. Data are from one biological repeat. LoD, limit of detection. Brackets indicate the Kan treatment period. (C, D) Shannon diversity index (C) and UniFrac (weighted and unweighted, as indicated) principle-coordinate analysis (D) of a 16S rRNA gene analysis of fecal samples from AIBC and nonpermissive mice at 0 and 45 DPI. Each point represents a mouse. Fecal samples from the same mice were analyzed at 0 and 45 DPI. Download FIG S4, TIF file, 0.6 MB. Copyright © 2021 Mullineaux-Sanders et al. 2021 Mullineaux-Sanders et al. https://creativecommons.org/licenses/by/4.0/ This content is distributed under the terms of the Creative Commons Attribution 4.0 International license.

To determine if there were any other changes to the gut microbiota of AIBC and nonpermissive mice, we performed 16S rRNA gene sequencing of fecal samples from AIBC and nonpermissive mice prior to infection (0 DPI) and during the AIBC/nonpermissive states (45 DPI). All groups showed comparable alpha diversities (Fig. S4C). Weighted principal-coordinate analysis (PCoA) showed separate clustering of the 0-DPI and 45-DPI mice, while unweighted PCoA additionally showed separate clustering of the AIBC and nonpermissive groups at 45 DPI (Fig. S4D). Nonpermissive mice showed a higher relative abundance of *Enterobacteriaceae* than AIBC mice, consistent with the observed colonization of nonpermissive mice by *C. amalonaticus* at levels approximately 100-fold higher than those of *C. rodentium* in AIBC mice. Notably, mice had relatively higher levels of S24-7 family members and relatively lower levels of *Anaeroplasmataceae* family members at 45 DPI compared to 0 DPI (Fig. 4C). However, these changes were similar between the AIBC and nonpermissive groups, suggesting that this did not impact colonization resistance to C. rodentium.

### Citrobacter amalonaticus governs permissibility to AIBC.

**FIG 5:**
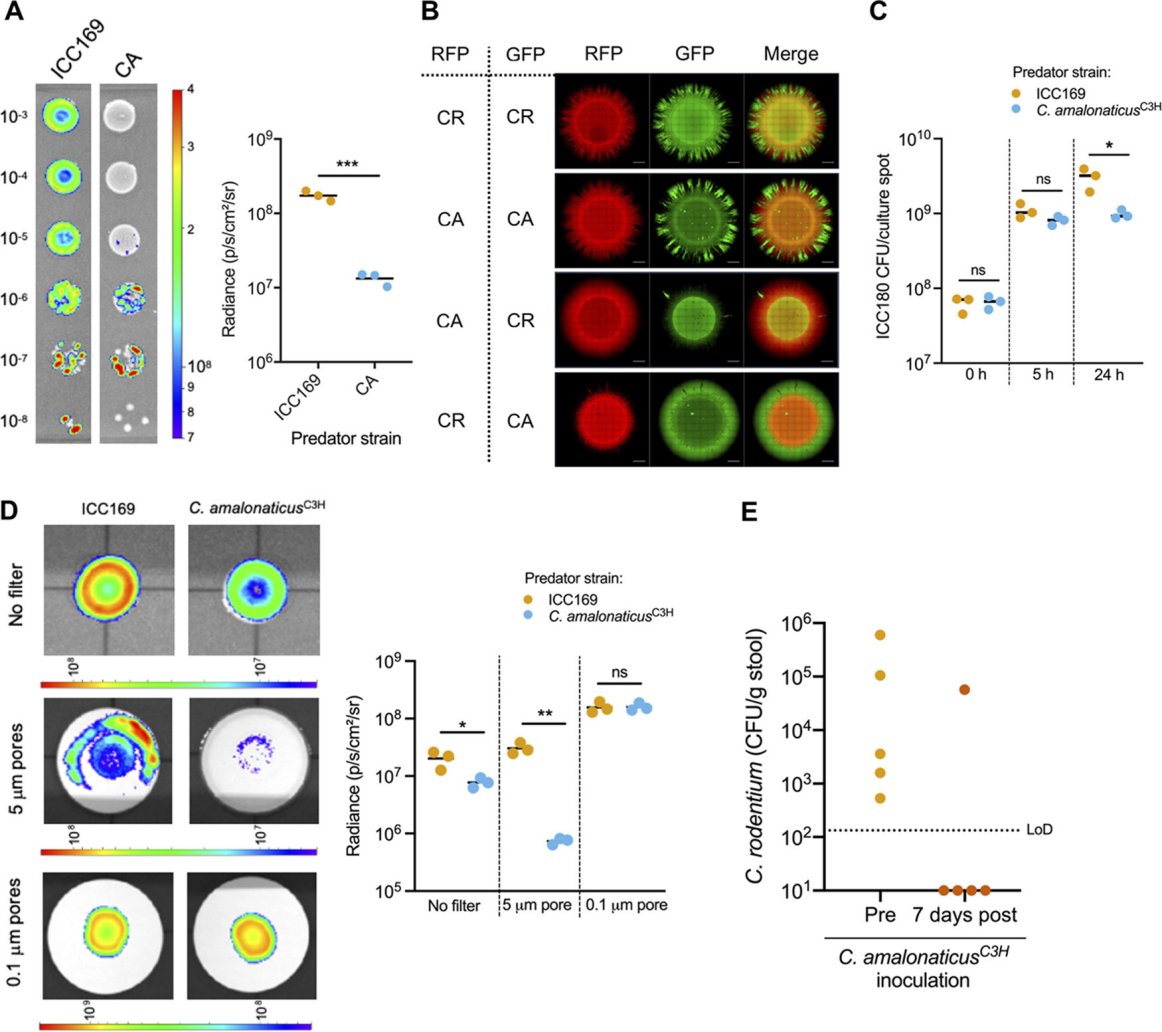
*C. amalonaticus*^C3H^ outcompetes C. rodentium. (A, left) Representative BLI of cocultures of ICC180 and ICC169 or *C. amalonaticus*^C3H^ (CA). The number on the left of the cultures indicates the dilution factor. (Right) Graph showing the quantification of BL from the 10^−3^ culture spot of the indicated coculture from three biological repeats. (B) Fluorescence microscopy of cocultures of C. rodentium (ICC169; CR) and *C. amalonaticus*^C3H^ (CA) expressing RFP or GFP from a plasmid as indicated after 24 h of growth on LB agar. Scale bar = 1,000 μm. (C) Quantification of ICC180 CFU at 0, 5, and 24 h after coincubation with ICC169 or *C. amalonaticus*^C3H^ (D, left) Representative BLI of ICC180 grown with ICC169 or *C. amalonaticus*^C3H^ separated by a filter with 5-μm or 0.1-μm pores or not separated by a filter. (Right) Graph showing the quantification of BL from the culture spots shown on the left from three biological repeats. (A, C, D) *, *P* < 0.05; **, *P* < 0.01; ***, *P* < 0.001 (as determined by Student’s unpaired two-tailed *t* test). (A, D) Color scale bars indicate radiance (photons per second per square centimeter per surface radiance). (E) C. rodentium colonization of AIBC mice before (Pre) *C. amalonaticus*^C3H^ inoculation (42 DPI) and 7 days after (post) the first *C. amalonaticus*^C3H^ inoculation (53 DPI). Five mice from one biological repeat were used. LoD, limit of detection.

We next tested the ability of *C. amalonaticus*^C3H^ to inhibit C. rodentium growth *in vitro*. Coculture on solid agar with *C. amalonaticus*^C3H^ inhibited C. rodentium ICC180 growth, as measured by a reduction in bioluminescence (BL) compared to levels after coculture with nonluminescent C. rodentium (ICC169) (Fig. 5A), in a temperature-independent manner (Fig. S5A). Further, fluorescence microscopy of cocultures of C. rodentium and *C. amalonaticus*^C3H^ expressing either green or red fluorescent protein (GFP or RFP, respectively) also showed inhibition of C. rodentium growth after 24 h (Fig. 5B; Fig. S5B). Quantification of C. rodentium ICC180 CFU at 0, 5, and 24 h postincubation with *C. amalonaticus*^C3H^ on LB agar (LBA) showed ICC180 growth between 0 and 5 h, similar to that observed when ICC180 was grown with ICC169 (nonbioluminescent C. rodentium); however, no further growth of ICC180 was observed between 5 and 24 h when cocultured with *C. amalonaticus*^C3H^, unlike with ICC180 cocultured with ICC169, which continued to grow (Fig. 5C). Together, these results suggest that *C. amalonaticus*^C3H^ inhibits C. rodentium growth at high bacterial densities but does not directly kill C. rodentium.

10.1128/mBio.02410-21.5 FIG S5 (A) BLI of cocultures of ICC180 and ICC169 or *C. amalonaticus*^C3H^ grown at the indicated temperature. Data are from three biological repeats. *, *P* < 0.05; **, *P* < 0.01; ***, *P* < 0.001 (as determined by Student’s unpaired two-tailed *t* test). (B) Cocultures of ICC169 (CR) and *C. amalonaticus*^C3H^ (CA) expressing RFP or GFP as indicated after 48 h of growth on LB agar. (C, left) Representative BLI of cocultures of ICC180 and ICC169, WT *C. amalonaticus*^C3H^, or *C. amalonaticus*^C3H^ lacking genes which encode proteins homologous to the T6SS Hcp (*ctsH4_2*), TssM (*00759*), or VgrG (*03332*) protein or RHS toxin-like protein (*wapA_4*) encoded adjacent to *00759*. The numbers above cultures indicate the dilution factor. Color scale bar indicates radiance (photons per second per square centimeter per surface radiance). (Right) Graph showing the quantification of BL from the 10^−3^ culture spot of the indicated culture from three biological repeats. ****, *P* < 0.001 (as determined by a one-way ANOVA with Tukey’s posttest for comparisons between all groups). (D) Colonization of C. rodentium, *C. amalonaticus*^C3H^, and *Escherichia*^C57^. C. rodentium colonization is shown for 3 individual mice, and *C. amalonaticus*^C3H^ and *Escherichia*^C57^ colonization is shown as the means ± SEM of colonization of the same 3 mice. Data are from one biological repeat. Inverted triangles indicate times of *Escherichia*^C57^ (blue triangles) and *C. amalonaticus*^C3H^ (green triangles) inoculations. (E) Colonization of C. rodentium and *C. amalonaticus*^C3H^. C. rodentium colonization is shown for 5 individual mice, *C. amalonaticus*^C3H^ colonization is shown as the mean ± SEM of colonization of the same 5 mice. Data are from one biological repeat. Inverted triangles show times of *C. amalonaticus*^C3H^ inoculations. (D, E) LoD, limit of detection. Download FIG S5, TIFF file, 1.6 MB. Copyright © 2021 Mullineaux-Sanders et al. 2021 Mullineaux-Sanders et al. https://creativecommons.org/licenses/by/4.0/ This content is distributed under the terms of the Creative Commons Attribution 4.0 International license.

Bioluminescent colonies of C. rodentium were observed at lower dilutions of the coculture with *C. amalonaticus*^C3H^, in which single colonies were resolved (Fig. 5A), suggesting that the growth inhibition may be contact dependent. To test this, C. rodentium ICC180 was cocultured on agar with either nonluminescent C. rodentium or *C. amalonaticus*^C3H^, with a 0.1-μm-pore-size filter placed between the cultures to allow the movement of nutrients and secreted products but to restrict direct contact between the bacteria. Filters with 5-μm pores (through which bacteria can traverse) or no filters were placed between the culture spots as controls. C. rodentium ICC180 showed significantly lower BL when grown with *C. amalonaticus*^C3H^ than when grown with nonluminescent C. rodentium when strains were separated by a 5-μm-pore filter or no filter (Fig. 5D). Conversely, similar bioluminescence was observed from spots of C. rodentium ICC180 grown on top of either nonluminescent C. rodentium or *C. amalonaticus*^C3H^ when the culture spots were separated by a 0.1-μm-pore filter (Fig. 5D), suggesting that the inhibition of C. rodentium growth by *C. amalonaticus*^C3H^ occurs in a contact-dependent manner.

Inspection of the genome sequence of *C. amalonaticus*^C3H^ did not reveal any obvious contact-dependent inhibition machinery (Table S1). While several genes encoding proteins homologous to proteins of the type VI secretion system (T6SS), commonly used in bacterial interspecies competition, were identified, *C. amalonaticus*^C3H^ lacked the full set of core components required to encode a functional T6SS (28). Indeed, *C. amalonaticus*^C3H^ strains with single deletions of genes encoding proteins homologous to the T6SS Hcp (*ctsH4_2*), TssM (*00759*), or VgrG (*03332*) components or RHS toxin-like protein (*wapA_4*), encoded adjacent to TssM, did not show a reduced ability to inhibit C. rodentium growth (Fig. S5C). Therefore, the molecular mechanism by which contact-dependent growth inhibition occurs requires further investigation.

10.1128/mBio.02410-21.6 TABLE S1 Annotation of the assembled *C. amalonaticus*^C3H^ genome. Download Table S1, XLSX file, 0.3 MB. Copyright © 2021 Mullineaux-Sanders et al. 2021 Mullineaux-Sanders et al. https://creativecommons.org/licenses/by/4.0/ This content is distributed under the terms of the Creative Commons Attribution 4.0 International license.

We next tested whether *C. amalonaticus*^C3H^ could reverse AIBC C. rodentium *in vivo*. A single colony, isolated from an uninfected C57BL/6 mouse and identified by 16S rRNA gene sequencing as the Escherichia*-Shigella* genus (referred to as *Escherichia*^C57^), was used as a control. In an initial experiment, 3 mice (harboring AIBC C. rodentium at 42 DPI and negative for *C. amalonaticus*^C3H^) were orally administered two inoculations of ∼1 × 10^9^ CFU *Escherichia*^C57^ (on DPI 42 and 44). *Escherichia*^C57^ persistently colonized the mice at a level similar to that of AIBC C. rodentium; however, this did not alter C. rodentium colonization up to 13 days after gavage (55 DPI) (Fig. S5D). These mice were subsequently orally administered two inoculations of *C. amalonaticus*^C3H^ (on DPI 55 and 57). *C. amalonaticus*^C3H^ colonized the mice at around 10^7^ CFU/GoF, similar to levels observed in naturally nonpermissive mice. Further, 2 of the 3 mice cleared C. rodentium 7 days following administration of *C. amalonaticus*^C3H^ (62 DPI) (Fig. S5D). Next, to ensure that this effect was a result of *C. amalonaticus*^C3H^ alone (rather than the combined effect of *Escherichia*^C57^ and *C. amalonaticus*^C3H^), 5 mice (harboring AIBC C. rodentium at 42 DPI and negative for *C. amalonaticus*^C3H^) were given two inoculations of *C. amalonaticus*^C3H^ (at 42 and 43 DPI). Again *C. amalonaticus*^C3H^ colonized at around 10^7^ CFU/GoF, and 4 of the 5 mice cleared C. rodentium 7 days following administration of *C. amalonaticus*^C3H^ (Fig. 5E; Fig. S5E). Overall, these results suggest that under the conditions of our model, the commensal *C. amalonaticus*^C3H^ is sufficient to confer colonization resistance to the growth of luminal C. rodentium.

## DISCUSSION

Enteric pathogens use both host- and microbiota-derived cues to control virulence gene expression and metabolism, which allows them to compete with and/or evade the microbiota and colonize their host (2, 29). Conversely, the gut microbiota assists the host in clearing invading pathogens through a process known as colonization resistance (17). Here, we identify the commensal bacterium *C. amalonaticus*^C3H^ as capable of inhibiting luminal C. rodentium growth *in vivo*. While previous studies have implicated commensal E. coli in outcompeting C. rodentium via nutrient deprivation (17), our study identifies an intragenus competition in which *C. amalonaticus*^C3H^ employs a contact-dependent mechanism to inhibit C. rodentium growth.

We find that a short Kan treatment course that induces taxonomic changes to the gut microbiota (14) and changes the anatomical localization of C. rodentium within the host results in the pathogen adopting a commensal lifestyle in C3H mice following cessation of the antibiotic treatment. The persistent growth of WT C. rodentium in the gut for long periods of time has previously been observed in germfree C57BL/6 mice (17) and in specific-pathogen-free (SPF) C57BL/6 mice under conditions of continuous Kan treatment (AIBP) (14). While it has been demonstrated that host diet alteration can select for genetically attenuated C. rodentium in C3H mice (26), we did not find evidence of acquisition of genetic mutations preventing T3SS function in WT C. rodentium in our luminal-colonization model. Conversely, C. rodentium harboring mutations in the LEE sequence were dominant following the 7-day Kan treatment period during infection with C. rodentium Δ*grlR*, which expresses these genes constitutively, suggesting that WT C. rodentium rapidly downregulates its LEE gene expression in the lumen, while the Δ*grlR* strain, which cannot modulate expression through regulation, achieves this through genetic mutation. While repression of *ler* in WT C. rodentium during AIBC remains to be directly shown, it has previously been demonstrated to occur during germfree mouse infection and during AIBP (14, 17). Understanding the signals which govern this virulence gene expression switch may provide a mechanism by which pathogen virulence can be attenuated *in vivo*.

Interestingly, despite persisting in the host following the withdrawal of Kan treatment, AIBC C. rodentium remains in the cecal luminal contents and does not recolonize the colonic mucosa, its physiological infection niche. It is possible that the cues required for LEE expression (and therefore mucosal colonization) are absent in the cecum following Kan treatment or that following the initial acute infection, the mucosa is refractory to recolonization by C. rodentium. During acute infection, LEE gene expression likely confers an advantage *in vivo* by allowing C. rodentium to colonize the mucosa, therefore allowing it to avoid competition with the luminal microbiota; however, expression is costly, and therefore under nonadvantageous conditions (e.g., the gut lumen) and in the absence of competitors, C. rodentium coexists with the host asymptomatically. It is notable that during Kan treatment (and during colonization of germfree mice [17]), C. rodentium colonizes at around 10^8^ to 10^9^ CFU/GoF, but during AIBC, this falls to around 10^5^ CFU/GoF, a colonization level previously reported for facultative anaerobic members of the microbiota (27). This suggests that 10^5^ CFU/GoF is the maximum C. rodentium colonization level that can be sustained in the context of the polymicrobial community of the cecal lumen.

We found that, following cessation of antibiotic treatment, a minority of C3H mice were refractory to the luminal growth of C. rodentium, suggesting that the microbiotas of these mice provided colonization resistance; we identified a commensal facultative anaerobe, *C. amalonaticus*^C3H^, which governs this. Similarly, differences in commensal species between genetically similar or identical mice have been reported to confer differences in susceptibility to Salmonella infection and dextran sulfate sodium (DSS)-induced colitis (27, 30). *C. amalonaticus*^C3H^ was not detected prior to infection and did not bloom until after withdrawal of the Kan treatment. Antibiotic depletion of the microbiota can create a niche favorable to the expansion of facultative anaerobes posttreatment by altering the nutrient and oxygen availability (31), which may have favored the expansion of *C. amalonaticus*^C3H^. For example, antibiotic treatment leads to increase oxygenation of the gut by killing butyrate-producing commensals (32, 33). Likewise, during acute infection with C. rodentium, IECs switch from ATP production in the mitochondria to aerobic glycolysis, which increases mucosal oxygenation and enables the expansion of closely related species (23). Notably, 20% of nonpermissive mice were also negative for *C. amalonaticus*^C3H^, which may indicate that there may be other commensals in these mice which are also able to provide colonization resistance against the luminal growth of C. rodentium in our model.

Commensal E. coli (strain dn15.6244.1) has previously been shown to outcompete avirulent luminal C. rodentium in germfree mice by competing for monosaccharides (17). Here, a C57BL/6 stool isolate of the Escherichia*-Shigella* genus was unable to outcompete luminal C. rodentium in conventional mice, despite robustly colonizing the gut, suggesting that, unlike with E. coli dn15.6244.1, the *Escherichia*^C57^ isolate does not overlap C. rodentium in its metabolic requirements. We find that *C. amalonaticus*^C3H^ inhibits C. rodentium growth *in vitro* in a contact-dependent manner, although we cannot rule out the possibility that other growth inhibition mechanisms, such as nutrient competition, may play a role *in vivo*. Contact-dependent killing via a T6SS contributes to commensal-pathogen competition during Vibrio cholerae (34), Shigella sonnei (35), and enterotoxigenic Bacteroides fragilis (36) murine gut infection. It is notable that *C. amalonaticus*^C3H^ colonizes at a ratio of approximately 100 to 1 C. rodentium in the lumen, which may allow effective contact-dependent inhibition in the gut lumen.

The line between a pathogenic and commensal organism is a thin one; many members of the microbiota are closely related to classically pathogenic organisms, and the virulence of many bacteria is environment dependent and costly if expressed under nonadvantageous conditions. C. rodentium is not a member of the murine gut microbiota; nevertheless, changes in the gut environment can prevent pathogenic behavior, favor downregulation of the LEE genes within the host, and drive it toward a commensal-like lifestyle. We show that commensals closely related to pathogenic bacteria can confer colonization resistance, preventing long-term carriage of enteric pathogens. In the future, growth inhibition by closely related commensals may be exploited to increase colonization resistance against bacterial pathogens to prevent acute infection.

## MATERIALS AND METHODS

### Bacterial strains and growth.

Bacterial strains used in this study are shown in Table S2 in the supplemental material (see also references 37 to 40). Bacteria were grown in lysogeny broth (LB; VWR) at 37°C and with 200-rpm shaking, where required. LB agar (LBA; VWR) was used for growth on solid medium. For cell culture infections, bacteria were grown in low-glucose Dulbecco’s modified Eagle’s medium (DMEM; Sigma) at 37°C and 5% CO_2_. Where required and unless otherwise specified, growth medium was supplemented with the following antibiotics at the indicated concentrations: kanamycin (Kan) at 50 μg/ml, nalidixic acid (Nal) at 50 μg/ml, streptomycin (Sm) at 50 μg/ml, rifamycin (Rif) at 50 μg/ml, gentamicin (Gm) at 10 μg/ml, and chloramphenicol (Cm) at 30 μg/ml.

10.1128/mBio.02410-21.7 TABLE S2 Bacterial strains used in this study. Download Table S2, PDF file, 0.04 MB. Copyright © 2021 Mullineaux-Sanders et al. 2021 Mullineaux-Sanders et al. https://creativecommons.org/licenses/by/4.0/ This content is distributed under the terms of the Creative Commons Attribution 4.0 International license.

### Animal experiments.

Animal experiments were performed in accordance with the Animals Scientific Procedures Act of 1986 (41) and UK Home Office guidelines and were approved by the Imperial College Animal Welfare and Ethical Review Body. Pathogen-free 8- to 10-week-old female C3H/HeN mice and pathogen-free C57BL/6 18- to 20-g female mice were purchased from Charles River Laboratories. Mice were housed in groups of 3 to 5 in individually HEPA-filtered cages with bedding, nesting, and free access to food and water. For each experiment, mice were randomly assigned to experimental groups. Investigators were not blind to the allocation. Mice which lost >20% of their starting weight or became moribund during the experimental period were culled and were excluded from all analyses except for the survival analysis. One mouse was culled for a separate welfare issue and was excluded from all analyses. Mice were infected with approximately 10^9^ CFU C. rodentium by oral gavage as previously described (15) or mock infected with 200 μl sterile phosphate-buffered saline (PBS). C. rodentium inoculum was retrospectively confirmed by serial dilution and quantification of CFU growth on LBA containing Kan. For Fig. 2, all mice were infected with C. rodentium strain ICC180, except for the Acute group mice, which were infected with ICC169. In all other experiments, mice were infected with the strains detailed in the text or figure legends. Where indicated, mice were orally gavaged once daily with Kan (20 mg in sterile water) or mock treated with 200 μl sterile water. For commensal inoculations, mice were inoculated with approximately 10^9^ CFU *C. amalonaticus*^C3H^ or *Esherichia*^C57^-Rif in 200 μl PBS, prepared as described for C. rodentium. Inoculum was retrospectively confirmed by serial dilution and quantification of CFU growth on LBA. For quantification of bacterial shedding, stools were resuspended in 10-ml/g sterile PBS and briefly pelleted (<2-s pulse) to remove large debris, and the supernatant was serially diluted and plated on LBA plus Kan or Nal (C. rodentium), LBA plus Rif (*Esherichia*^C57^-Rif), or unsupplemented LBA (*C. amalonaticus*^C3H^). For *C. amalonaticus*^C3H^ quantification, *Citrobacter* colonies were identified by eye based on their colony morphology, and *C. amalonaticus*^C3H^ was subsequently distinguished from C. rodentium ICC180 by bioluminescence imaging (BLI) of the agar plates and exclusion of bioluminescent colonies. Ten colonies identified as *C. amalonaticus*^C3H^ were further confirmed by 16S rRNA gene sequencing, which indicated a 100% accurate identification.

### Designation of AIBC and nonpermissive mice.

AIBC mice were defined as C3H/HeN mice which, following ICC180 infection and Kan treatment from 6 to 12 DPI, harbored fecal C. rodentium ICC180 (detectable by plating on LB agar plus 50 μg/ml Kan at 63 DPI, above the detection threshold of 1.3 × 10^2^ CFU/GoF) without more than one C. rodentium-negative sample during the experimental period (until 63 DPI or, where relevant, until mice were inoculated prior to 63 DPI with a commensal). Nonpermissive mice were defined as C3H/HeN mice which, following ICC180 infection and Kan treatment from 6 to 12 DPI, became negative for C. rodentium during the experimental period and did not have any subsequent C. rodentium-positive stool samples. At least 2 consecutive C. rodentium-negative stools samples were obtained at least 7 days apart from nonpermissive mice prior to experiment termination (nonpermissive mice were culled at 34 or 63 DPI). The line graph in Fig. 1C shows *C. rodentium* colonization of AIBC mice followed until 63 DPI without commensal inoculation only; however, all mice designated AIBC (including those subsequently inoculated with commensal species) are included in the pie chart.

### BLI.

Two-dimensional (2D) bioluminescent imaging of mice, tissue, and agar plates was performed using an IVIS Spectrum CT system (Perkin Elmer). For whole-animal imaging, mice were depilated on their abdomens using hair removal cream prior to the imaging and were maintained under gaseous anesthesia with isoflurane (Zoetis). For tissue imaging, gastrointestinal tissues were excised, the colon was cut longitudinally, the stool was removed with tweezers, the cecal contents were gently removed from the cecal tissue, and the cecal tissue washed briefly in PBS. All tissue was imaged with the mucosa exteriorized. BLI images were processed using the LivingImage software (v4.3.1), and radiance was quantified using the region of interest (ROI) tool.

### LCN-2 ELISA.

Stool samples were homogenized in 10 ml/g PBS with 0.1% Tween 20 using a vortex machine for 15 min. Samples were centrifuged at 16,000 rpm for 10 min, and the supernatant was extracted and stored at −80°C. The LCN-2 concentration was determined using a DuoSet mouse lipocalin-2 enzyme-linked immunosorbent assay (ELISA) (R&D Systems) according to the manufacturer’s instructions.

### Histological analysis and immunostaining.

Formalin-fixed 0.5-cm whole distal colon, proximal colon, or distal ileum samples were processed, paraffin embedded, and sectioned at 5 μm. Formalin-fixed, paraffin-embedded (FFPE) sections were then either stained with hematoxylin and eosin (H&E) by standard techniques or processed for immunofluorescence. For immunofluorescence, sections were dewaxed by submersion in Histo-Clear solution (VWR) twice for 10 min, 100% ethanol twice for 10 min, 95% ethanol twice for 3 min, 80% ethanol once for 3 min, and PBS–0.1% Tween 20–0.1% saponin (PBS-TS) twice for 3 min. Subsequently, sections were heated for 30 min in demasking solution (0.3% trisodium citrate, 0.05% Tween 20 in distilled H_2_O). Slides were blocked in PBS-TS supplemented with 10% normal donkey serum (NDS; ThermoFisher Scientific) for 20 min in a humid chamber and then incubated with polyclonal rabbit anti-C. rodentium O152 (1:50; Statens Serum Institute, Copenhagen, Denmark) and mouse anti-E-Cadherin antibody (1:50; CD324, BD Biosciences) in PBS-TS-NDS for 1 h. Slides were washed twice for 10 min in PBS-TS, followed by incubation with donkey anti-mouse Alexa Flour 488 (Jackson Immunoresearch; 1:100) and donkey anti-rabbit Alexa Flour 555 (Jackson Immunoresearch; 1:100) and Hoechst 33342 stain (1:1,000 dilution). Slides were mounted with ProLong Gold antifade mountant (Thermo Fisher Scientific). Images were acquired using a Zeiss AxioVision Z1 microscope equipped with an AxioCam 105 color camera or Hamamatsu ORCA-Flash 4.0 C11440 camera and processed using Zen 2.3 Blue Version (Carl Zeiss MicroImaging GmbH, Germany). CCH measurements were performed on H&E-stained sections and were obtained from at least 10 well-oriented crypts per mouse.

### IEC purification.

Colonic IECs were isolated from 3.5-cm distal colonic tissue (following removal of the most distal 0.5-cm tissue for histological analysis), as previously described (24). Briefly, colonic tissue was opened longitudinally and briefly washed in 1× Hanks’ balanced salt solution (HBSS) without Mg and Ca. The tissue was incubated at 37°C with shaking for 45 min in enterocyte dissociation buffer (1× HBSS without Mg and Ca, containing 10 mM HEPES, 1 mM EDTA, and 5 μl/ml 2-β-mercaptoethanol). The remaining tissue was removed, and the lifted enterocytes were subsequently collected by centrifugation (2,000 × *g* for 10 min), followed by three PBS washes at 4°C. Enterocyte pellets were stored at −80°C.

### qRT-PCR.

RNA was isolated from purified IECs using the RNeasy minikit (Qiagen) according to the manufacturer’s instructions. RNA was treated with RQ1 RNase-free DNase (Promega) for 30 min, and subsequently cDNA was synthesized using a Moloney murine leukemia virus reverse transcription kit (Promega). Amplification from cDNA was performed using Power SYBR green PCR master mix (ThermoFisher Scientific). Assay reactions were performed in a 20-μl volume with 2× Power SYBR green PCR master mix with efficiency-optimized primers to a final concentration of 0.05 μM each and 1 to 10 ng total cDNA. All reactions were carried out in technical duplicates. The ΔΔ*C_t_* method (where *C_t_* is threshold cycle) of quantification was performed to give the log_2_ fold change from the expression of averaged baseline measurements (42). Expression was normalized to that of the housekeeping gene *Gapdh*. Primers used are listed in Table S3.

10.1128/mBio.02410-21.8 TABLE S3 Primers used in this study. Download Table S3, TIF file, 0.9 MB. Copyright © 2021 Mullineaux-Sanders et al. 2021 Mullineaux-Sanders et al. https://creativecommons.org/licenses/by/4.0/ This content is distributed under the terms of the Creative Commons Attribution 4.0 International license.

### C. rodentium infections of CMT-93 cells.

The mouse colonic cell line CMT-93 was grown in high-glucose DMEM (Sigma) supplemented with 10% fetal bovine serum (Gibco), 2 mM glutamine (Gibco), and 1% MEM nonessential amino acids (Sigma) at 37°C with 5% CO_2_. For infections, cells were seeded on glass coverslips in 24-well plates at a concentration of 5 × 10^4^ cells/well and grown for 48 h. Saturated LB C. rodentium cultures were diluted 1:100 in low-glucose DMEM (Sigma) and grown overnight at 37°C and 5% CO_2_. One hundred microliters of overnight culture was added to each well. Plates were centrifuged for 5 min at 500 × *g* and incubated at 37°C and 5% CO_2_ for 3 h. Cells were then washed three times with sterile PBS, fixed with 4% (wt/vol) paraformaldehyde in PBS for 15 min, quenched with 50 mM NH_4_Cl, and permeabilized in 0.1% (vol/vol) Triton X-100 in PBS for 5 min. Coverslips were blocked in PBS supplemented with 1% bovine serum albumin (BSA) for 1 h and then incubated with polyclonal rabbit anti-C. rodentium O152 (1:100; Statens Serum Institute, Copenhagen, Denmark) in PBS-BSA for 1 h. Coverslips were washed three times in PBS, followed by incubation with Donkey anti-rabbit Alexa Flour 488 (Jackson Immunoresearch; 1:200), Phallodin-iFlour 647 (Stratech; 1:100), and Hoechst 33342 dye or 4′,6-diamidino-2-phenylindole (DAPI) (1:1,000) for 1 h. Coverslips were washed three times in PBS and once in distilled H_2_O and were mounted with ProLong Gold antifade mountant (Thermo Fisher Scientific). Images were acquired using a Zeiss AxioVision Z1 microscope equipped with a Hamamatsu ORCA-Flash 4.0 C11440 camera and processed using Zen 2.3 (Blue Version) (Carl Zeiss MicroImaging GmbH, Germany).

### Commensal bacterial isolation and identification.

To isolate commensal bacteria, stool samples were resuspended in 10 ml/g sterile PBS, plated on unsupplemented LBA, and grown at 37°C overnight under atmospheric conditions. C. rodentium ICC180 colonies were identified by BLI of the plates, and bioluminescent colonies were excluded. Colonies were grouped based on their colony morphology and subsequently identified by 16S rRNA gene sequencing of a minimum of 12 colonies/group. For 16S rRNA gene sequencing, individual colonies were resuspended in sterile water containing glass beads and heated at 95°C for 5 min, followed by manual cell disruption using a vortex machine for 10 min. Cell debris was pelleted by brief-pulse centrifugation, and 1 to 5 μl supernatant used as the template for PCR with primers 16S_27F and 16S_1510R using OneTaq 2× master mix (NEB), performed according to the manufacturer’s instructions. PCR cleanup and DNA sequencing of the amplified product with the 16S_27F primer was performed by Eurofins. 16S rRNA gene sequences generated from the 16S_27F primer were used to identify the colony genus using the SILVA rRNA gene database “search and classify” function (43). A single isolate of the *Citrobacter* genus was subjected to whole-genome sequencing.

### *C. amalonaticus*^C3H^ whole-genome sequencing.

*C. amalonaticus*^C3H^ DNA was isolated using a MasterPure Complete DNA and RNA purification kit (Lucigen). The genome was sequenced on the Illumina HiSeq X 10 platform and Nanopore GridION. Long reads were demultiplexed using qcat v1.1.0 (https://github.com/nanoporetech/qcat) and filtered by quality at a minimal Phred score of 10 using NanoFilt v2.5.0 (44). A merged assembly was generated using Unicycler pipeline v0.4.7 (45), with the high-quality long reads and the properly paired reads mapped to an initial version of a long-read assembly generated with Canu v1.6 (46) and Pilon v1.23 (5 rounds) (47). This resulted in a high-quality finished genome that was 4,847,284 bp in length and consisted of one circularized contig. Completeness and contamination were 99.97 and 0.04, respectively, according with CheckM v1.1.2 results (48). Following assembly, the genome was annotated using annotation pipelines at the Wellcome Sanger Institute (49), consisting of gene assignment using PROKKA v1.5 (50) and assignment of gene function by comparison to those of the Citrobacter amalonaticus genome from RefSeq (50, 51).

### Phylogenetic analysis.

Sequences of full-length 16S rRNA genes were obtained for selected *Citrobacter* species from the SILVA rRNA gene database (43) or RefSeq (51) for comparison to the full-length 16S rRNA gene of *C. amalonaticus*^C3H^. A consensus sequence from multiple 16S rRNA copies within a strain was obtained using the EMBL-EMI EMBOSS-Cons tool (52). Phylogenetic analysis was conducted in MEGA v7.0 (53). The evolutionary history was inferred by using the maximum likelihood method based on the Tamura-Nei model (54). Figure 4B shows the tree with the highest log likelihood (−2851.28). Initial tree(s) for the heuristic search were obtained automatically by applying the Neighbor-Join and BioNJ algorithms to a matrix of pairwise distances estimated using the maximum composite likelihood (MCL) approach and then selecting the topology with the superior log likelihood value. The analysis involved 10 nucleotide sequences. All positions containing gaps and missing data were eliminated. There were a total of 1,521 positions in the final data set.

### 16S rRNA gene sequencing.

DNA was extracted from frozen stool samples using a DNeasy PowerSoil kit (Qiagen). The 16S V4 region was amplified with the 515F and 806R primers. Amplicons were subsequently indexed using the Nextra XT set A (Illumina) primers and sequenced on an Illumina MiSeq with 2× 300-bp paired-end reads. Samples were demultiplexed based on sample-specific indices using Illumina RTA software (v1.18.54). Sequences were analyzed using the Qiime2 (version 2020.8) analysis pipeline (55). Demultiplexed FASTA-quality files were used as inputs. Reads were denoised, low-quality reads trimmed, paired-end reads assembled into longer reads, and sequences binned into amplicon sequencing variants using the Dada2 plugin for Qiime2 (56). Alpha and beta diversity analyses were performed using the q2-phylogeny and q2-diversity plugins. Taxonomy was assigned using a naive Bayes classifier trained against the Greengenes 13_8 database, trimmed to contain only the V4 region hypervariable region and preclustered at 99% sequence identity. For taxonomic analysis, any sequences not assigned to the phylum level were excluded.

### MIC testing.

The MIC of Kan for *C. amalonaticus*^C3H^ was determined by an agar dilution MIC, performed in accordance with published guidance (57). A saturated overnight culture of *C. amalonaticus*^C3H^ was diluted in PBS, and 20 μl (containing 10^4^ to 10^5^ CFU) was plated on Mueller-Hinton agar (Merck, UK) supplemented with Kan and incubated overnight at 37°C.

### Generation of spontaneous antibiotic-resistant commensal mutants.

*C. amalonaticus*^C3H^ was isolated from the stool of a nonpermissive C3H/HeN mouse at 41 DPI. *Escherichia*^C57^ was isolated from the stool of an uninfected C57BL/6 mouse. A spontaneous Sm-resistant mutant of *C. amalonaticus*^C3H^ (*C. amalonaticus*^C3H^-SmR) was obtained by sequentially passaging *C. amalonaticus*^C3H^ on LBA supplemented with increasing concentrations of Sm (10 to 100 μg/ml). *C. amalonaticus*^C3H^ was then passaged once on unsupplemented LBA before growing once more on LBA supplemented with 100 μg/ml Sm to ensure selection of a stable Sm-resistant mutant. A spontaneous Rif-resistant mutant of *Escherichia*^C57^ (*Escherichia*^C57^-Rif) was obtained as described above using Rif concentrations of 5 to 100 μg/ml.

### Electroporation of *Citrobacter* strains.

Unless otherwise specified, plasmids were introduced into C. rodentium and *C. amalonaticus*^C3H^ via electroporation. Briefly, log-phase bacteria were washed twice and resuspended in 15% glycerol. Approximately 50 to 100 ng of plasmid DNA was added to the cells, and a 2.5-kV pulse was applied (300-Ω resistance, 25-μF capacitance) to electrocompetent bacteria in 0.2-cm cuvettes through a GenePulser II (Bio-Rad). Bacteria were recovered at 37°C from 200-rpm LB for 1 h and then plated on LBA with appropriate antibiotics to select for transformants.

### Construction of gene deletions in *C. amalonaticus*^C3H^.

All gene deletions were constructed on the *C. amalonaticus*^C3H^-SmR background. All primers used in this study are listed in Table S3. All plasmids used in this study are shown in Table S4 (see also references 58 to 60). Homology regions (HRs) consisting of 500 bp upstream and the first three codons of the gene of interest (GOI; “Up HR”) and 500 bp downstream, including the last three codons of the GOI (“Down HR”) were amplified by PCR using the primer pairs …UPHR_F/…UPHR_R and …DNHR_F/…DNHR_R, respectively, with Q5 high-fidelity 2× MasterMix (NEB), according to the manufacturer’s instructions. pSEVA612S was linearized using primers pSEVA612S_F and pSEVA612S_R and joined with the Up and Down HRs using the Gibson Assembly Mastermix (NEB) according to the manufacturer’s instructions to create mutator plasmids for individual GOIs. Correct insertion of the of the up and down HRs into the pSEVA612S plasmid was confirmed by Sanger DNA sequencing (Eurofins) with M13 primers. For gene *03332*, a construct of the up and down HRs was synthesized (ThermoFisher) with flanking BamHI- and EcoRI-cut sites and subsequently digested with BamHI and EcoRI (NEB) according to the manufacturer’s instructions and then ligated into pSEVA612S digested with BamHI and EcoRI with T4 ligase (NEB) according to the manufacturer’s instructions. *C. amalonaticus*^C3H^ gene deletions were created by a two-step recombination process with the relevant pSEVA612S mutator plasmid. pSEVA612S mutator plasmids were introduced to *C. amalonaticus*^C3H^ via tri-parental conjugation. Briefly, 20 μl of a helper strain, E. coli 1047 pRK2013, was incubated with 20 μl of the donor strain, E. coli CC118-λ*pir* pSEVA612S, for 2 h at 37°C on LBA. Forty microliters of the receiver strain (*C. amalonaticus*^C3H^ harboring pACBSR) was added, and the plate was incubated for 4 h at 37°C. Conjugants were selected on LBA+Gm+Cm. Conjugants were grown in LB+Cm, supplemented with 0.4% l-arabinose for 8 h to induce expression of the I-SceI endonuclease from pACBSR, and subsequently streaked on LB+Sm plates. Cm^r^ Gm^s^ colonies (i.e., those in which the pSEVA612S plasmid backbone had successfully been removed) were selected and colonies screened for confirmation of the gene deletion using primers …Check_F/…Check_R by PCR with 2× OneTaq Mastermix (NEB) according to the manufacturer’s instructions. pACBSR was subsequently removed by passaging strains several times in LB, and bacteria sensitive to Cm were selected.

10.1128/mBio.02410-21.9 TABLE S4 Plasmids used in this study. Download Table S4, PDF file, 0.03 MB. Copyright © 2021 Mullineaux-Sanders et al. 2021 Mullineaux-Sanders et al. https://creativecommons.org/licenses/by/4.0/ This content is distributed under the terms of the Creative Commons Attribution 4.0 International license.

### C. rodentium competition assays.

For competition assays performed on solid medium, bacterial strains were prepared by growing single-strain cultures overnight in 5 ml LB supplemented with the appropriate antibiotics at 37°C with 200-rpm shaking. One milliliter of overnight culture was pelleted at 14,000 rpm for 1 min, the supernatant was removed, and the pellet was resuspended in 1 ml fresh LB without antibiotics. For all strains used, this results in a starting culture of approximately 5 × 10^9^ CFU/ml. For each assay, the starting culture density was retrospectively confirmed by serial dilution in PBS and plating on LB containing the appropriate antibiotics.

For bioluminescence competition assays, C. rodentium ICC180 was mixed 1:1 with the indicated competitor strain and briefly vortexed, and the mixed culture was serially diluted in sterile PBS and 25 μl of the 10^−3^ to 10^−8^ dilutions plated in triplicate on LBA (no antibiotics). Plates were incubated for 24 h at the indicated temperature and then subjected to BLI. For contact-dependent assays, 40 μl (undiluted) competitor strain (C. rodentium ICC169 or *C. amalonaticus*^C3H^) was plated onto LBA and allowed to dry. A 25-mm cellulose nitrile filter with 5-μm or 0.1-μm pores (Whatman) was placed over the culture spot, and 20 μl C. rodentium ICC180 (2:1 competitor-to-ICC180 ratio) was placed on top in the center of the filter (or directly on top on the culture spot for the no-filter control). The ICC180 spot was allowed to dry, and plates were incubated for 24 h at 37°C under atmospheric conditions.

For fluorescence competition assays, competitor strains were mixed in a 1:1 ratio and briefly vortexed, and 2 μl of the mixed culture was plated on LBA+Gm (for maintenance of the fluorophore-expressing pULTRA plasmid). Cultures were incubated at 37°C under atmospheric conditions, and fluorescence images were acquired after 24 h using a Zeiss AxioVision Z1 microscope, processed using the Zen 2.3 blue version (Carl Zeiss MicroImaging GmbH, Germany), and photographed after 48 h of growth.

For quantification of ICC180 growth in cocultures, ICC180 (undiluted) was mixed 1:1 with the indicated competitor strain and 20 μl of the mixed culture placed on top of a 25-mm cellulose nitrile filter with 0.1-μm pores (Whatman) on a LBA plate. At the indicted time, the filter was removed from the agar plate and placed into a 1.5-ml Eppendorf tube with 1 ml LB and vortexed for 30 s to dislodge bacteria from the filter into the media, which was subsequently serially diluted in sterile PBS and plated on LB+Kan to select for ICC180.

### Statistical analysis and data presentation.

Data were plotted and statistically analyzed in Prism (version 8). Survival curves were analyzed by a log rank (Mantel-Cox) test. Other data which passed a Kolmogorov-Smirnov or a Shapiro-Wilk normality test were analyzed using a Student unpaired two-tailed *t* test or a one-way ANOVA with Tukey’s posttest for comparisons between all groups, as specified. *P* values of <0.05 were considered significant. Where indicated, outliers were identified by the ROUT method. Data that did not pass the specified normality test following identification and removal of outliers were analyzed by a Kruskal-Wallis test with Dunn’s posttest for multiple comparisons between all groups. Schematic figures were created using BioRender (BioRender.com).

### Data exclusions.

Mice which were culled before the experimental endpoint for welfare reasons were excluded from all analysis except for survival analysis (as detailed in “Animal experiments” above). Two mice used as part of this study to test the effect of *C. amalonaticus*^C3H^ inoculation in mice harboring AIBC C. rodentium (Fig. S5D) were retrospectively found to have a C. rodentium-negative stool sample on the day of intervention with commensal inoculation and were subsequently excluded from all analysis, with the exception of the survival curve analysis. No other mice were excluded from this study.

### Data accessibility.

The genome assembly of *C. amalonaticus*^C3H^ has been deposited in the NCBI database with the accession number CP080958.
