# Supplementary figures and images for: Citrobacter amalonaticus Inhibits the Growth of Citrobacter rodentium in the Gut Lumen
Source: mBio. 2021 Oct 5;12(5):e02410-21. doi: 10.1128/mBio.02410-21 (PMC8510533; doi:10.1128/mBio.02410-21)

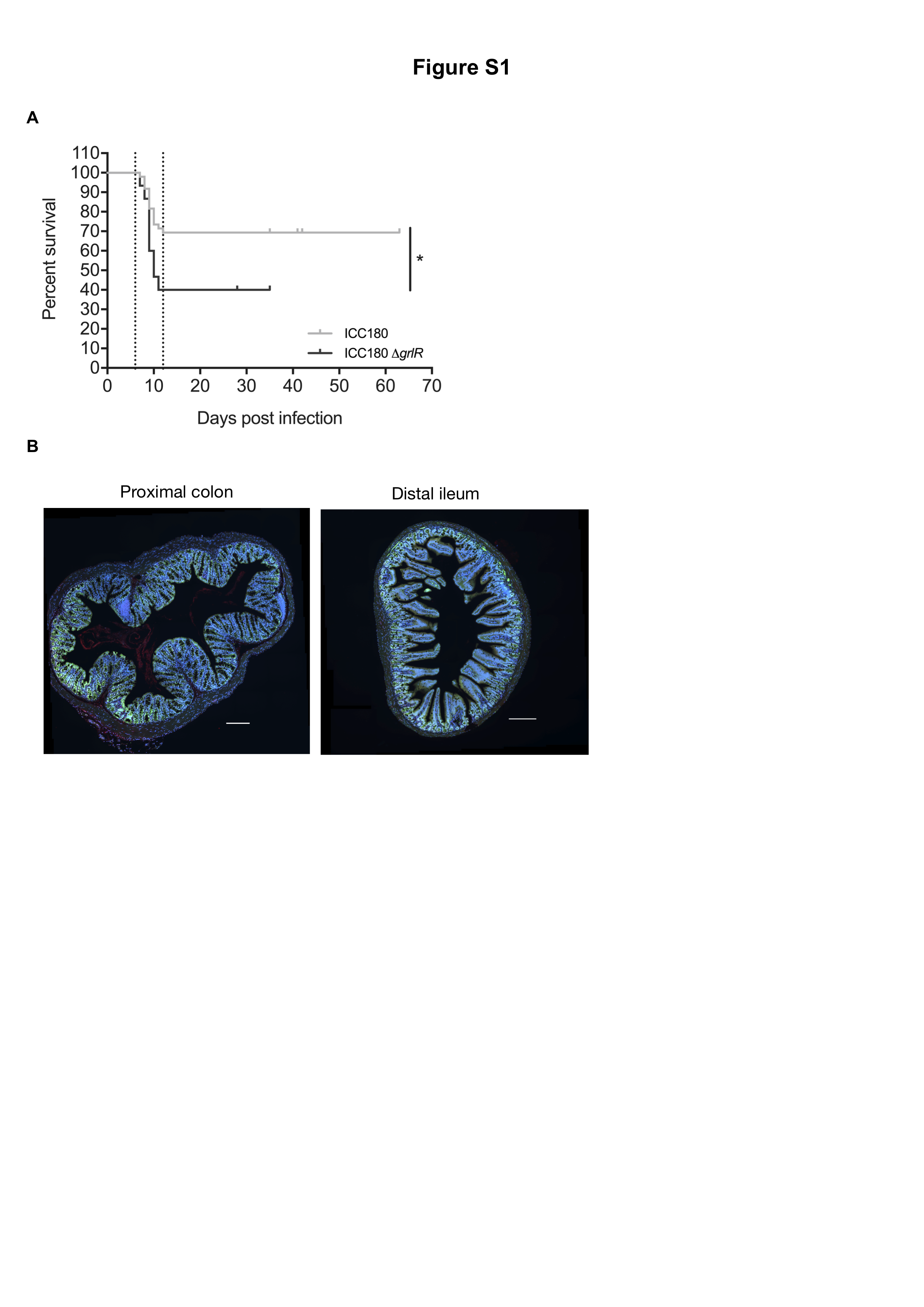

Supplement: FIG S1 [file mbio.02410-21-sf001.tif]

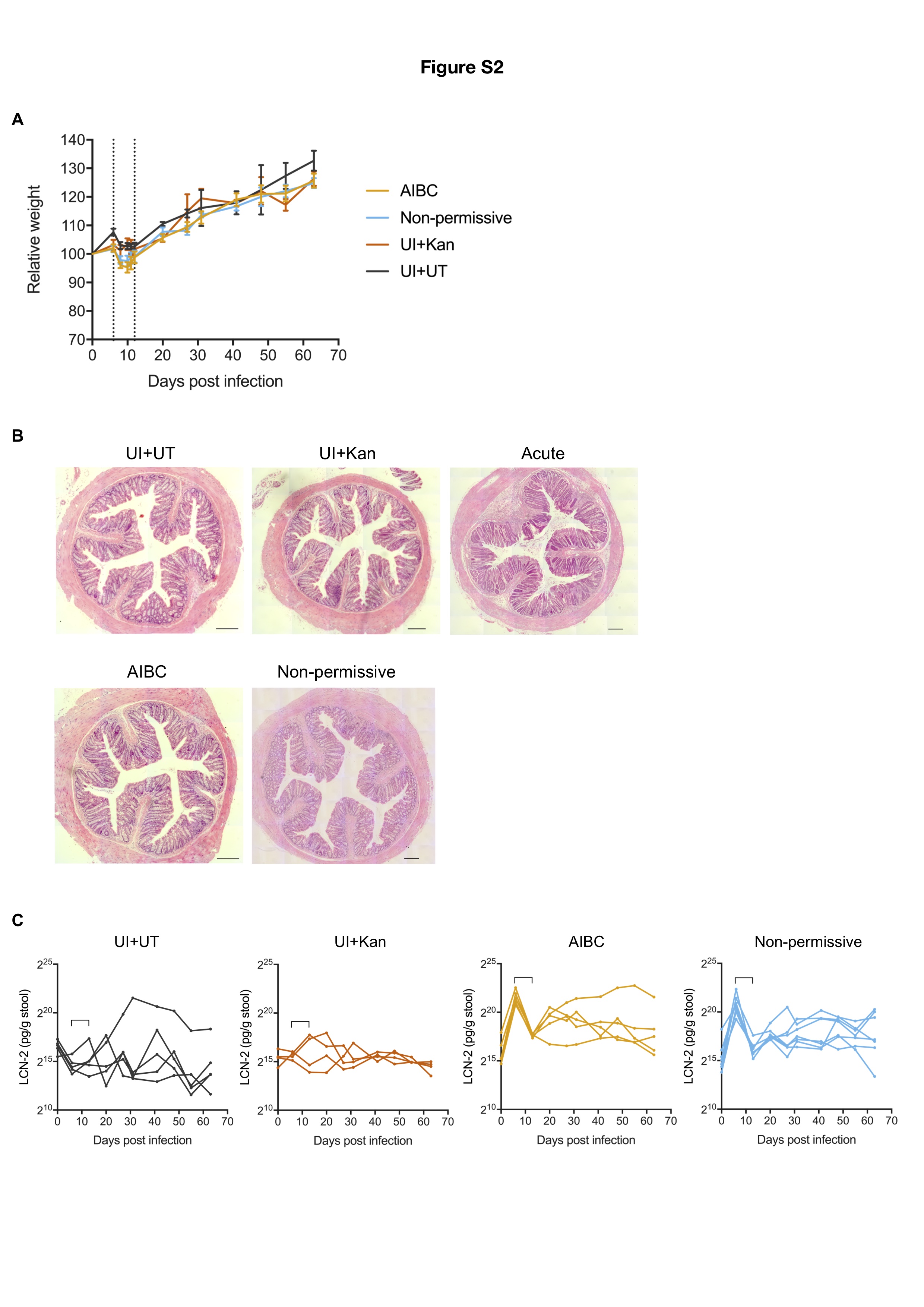

Supplement: FIG S2 [file mbio.02410-21-sf002.jpg]

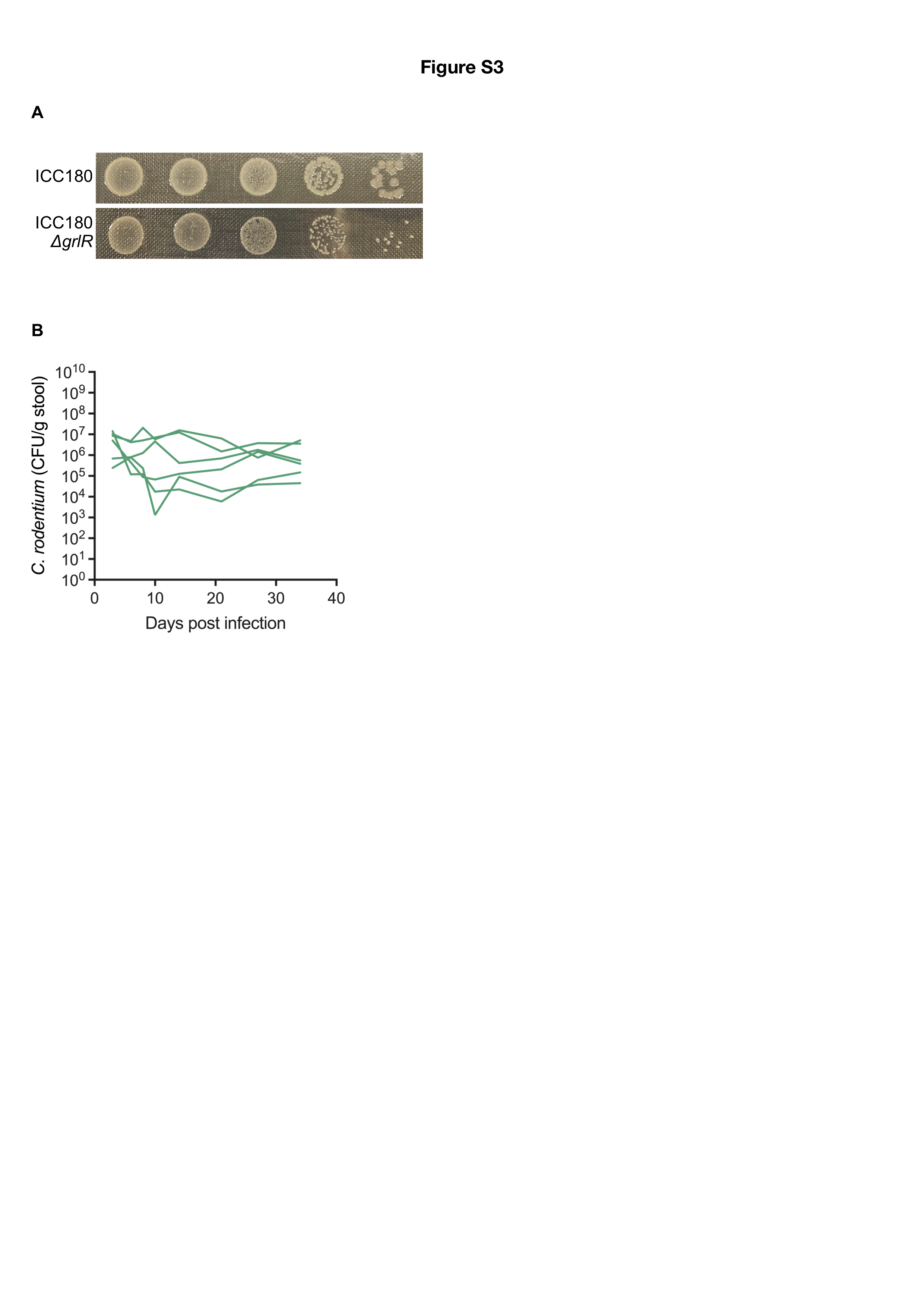

Supplement: FIG S3 [file mbio.02410-21-sf003.tiff]

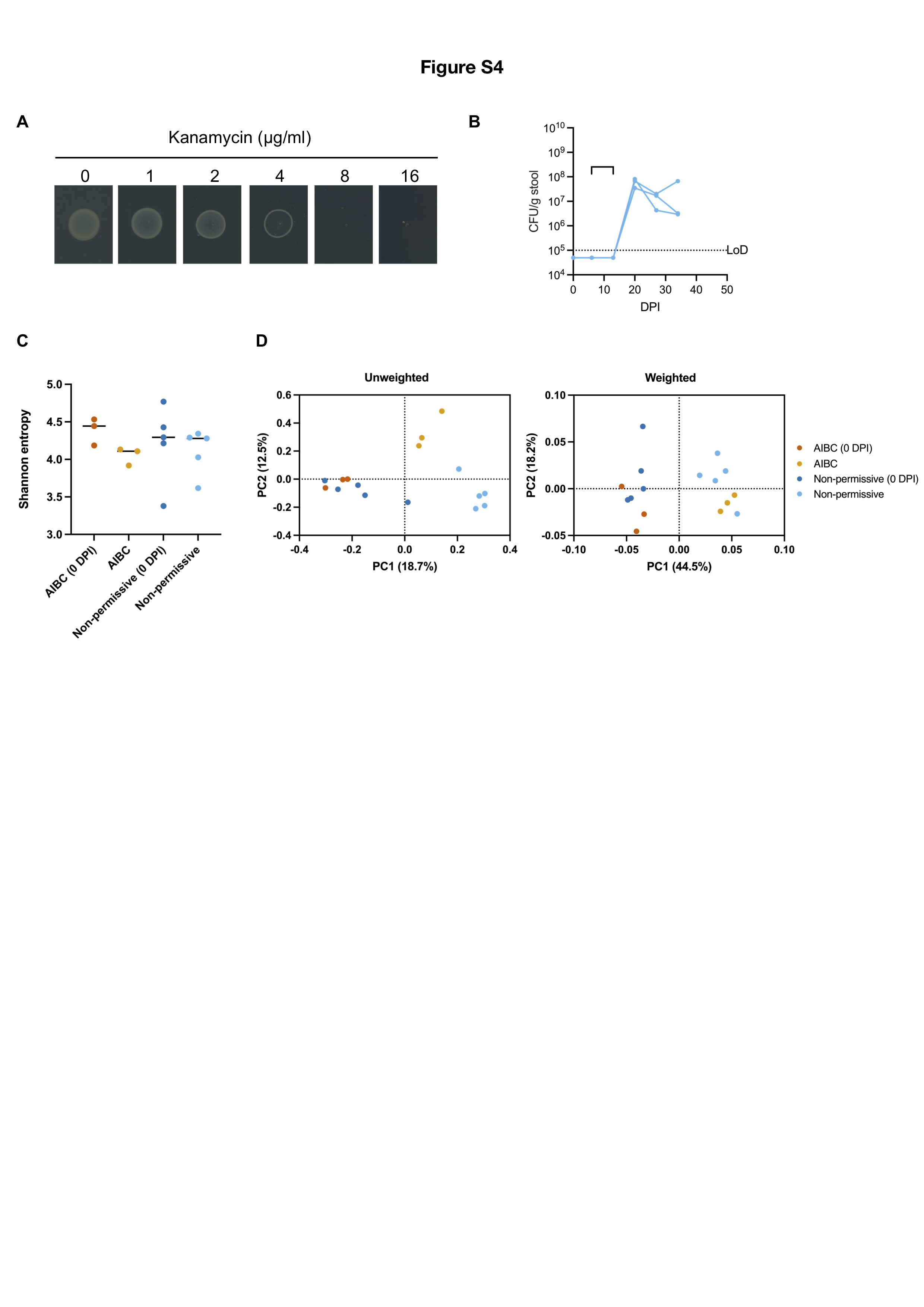

Supplement: FIG S4 [file mbio.02410-21-sf004.tif]

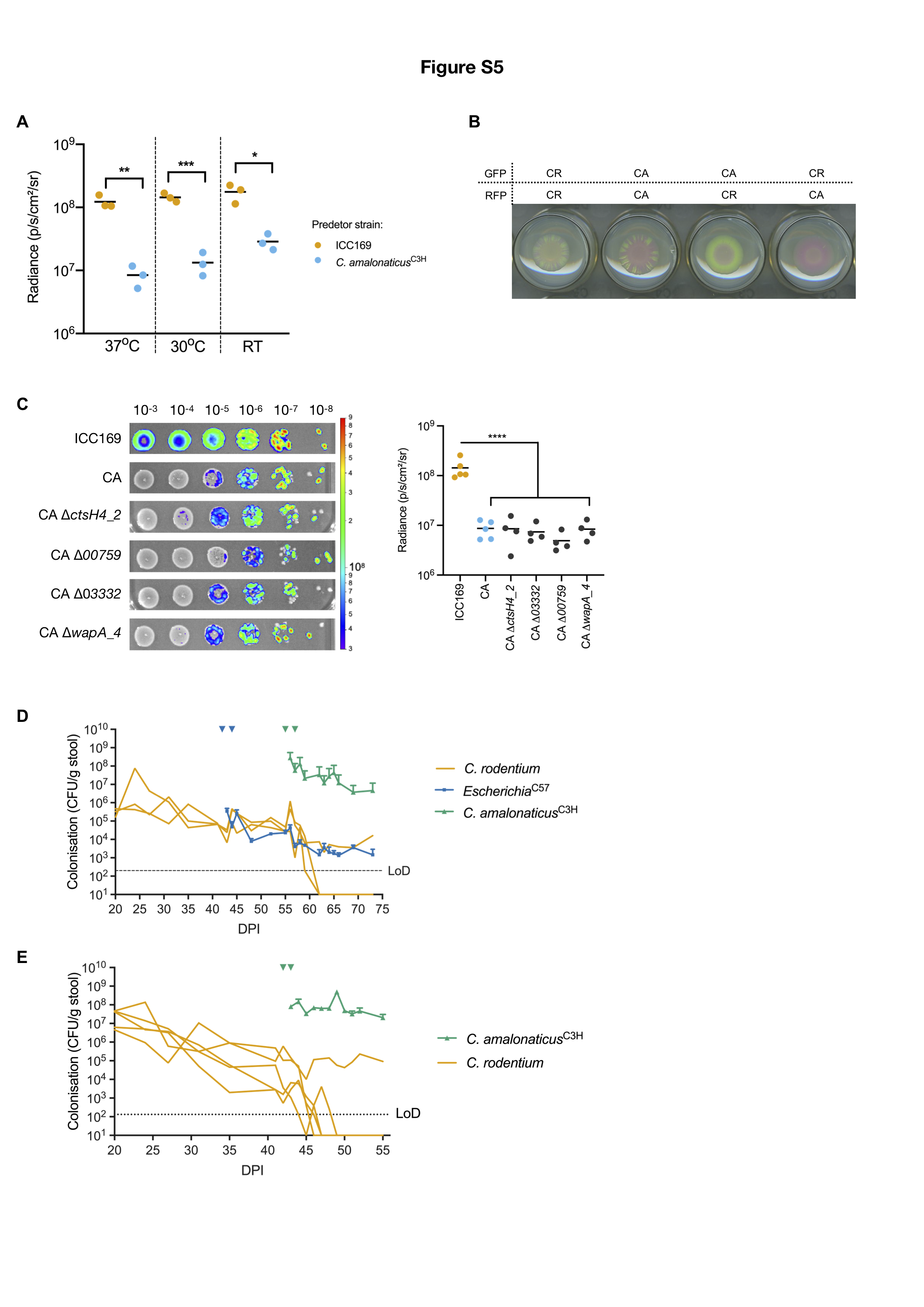

Supplement: FIG S5 [file mbio.02410-21-sf005.tiff]

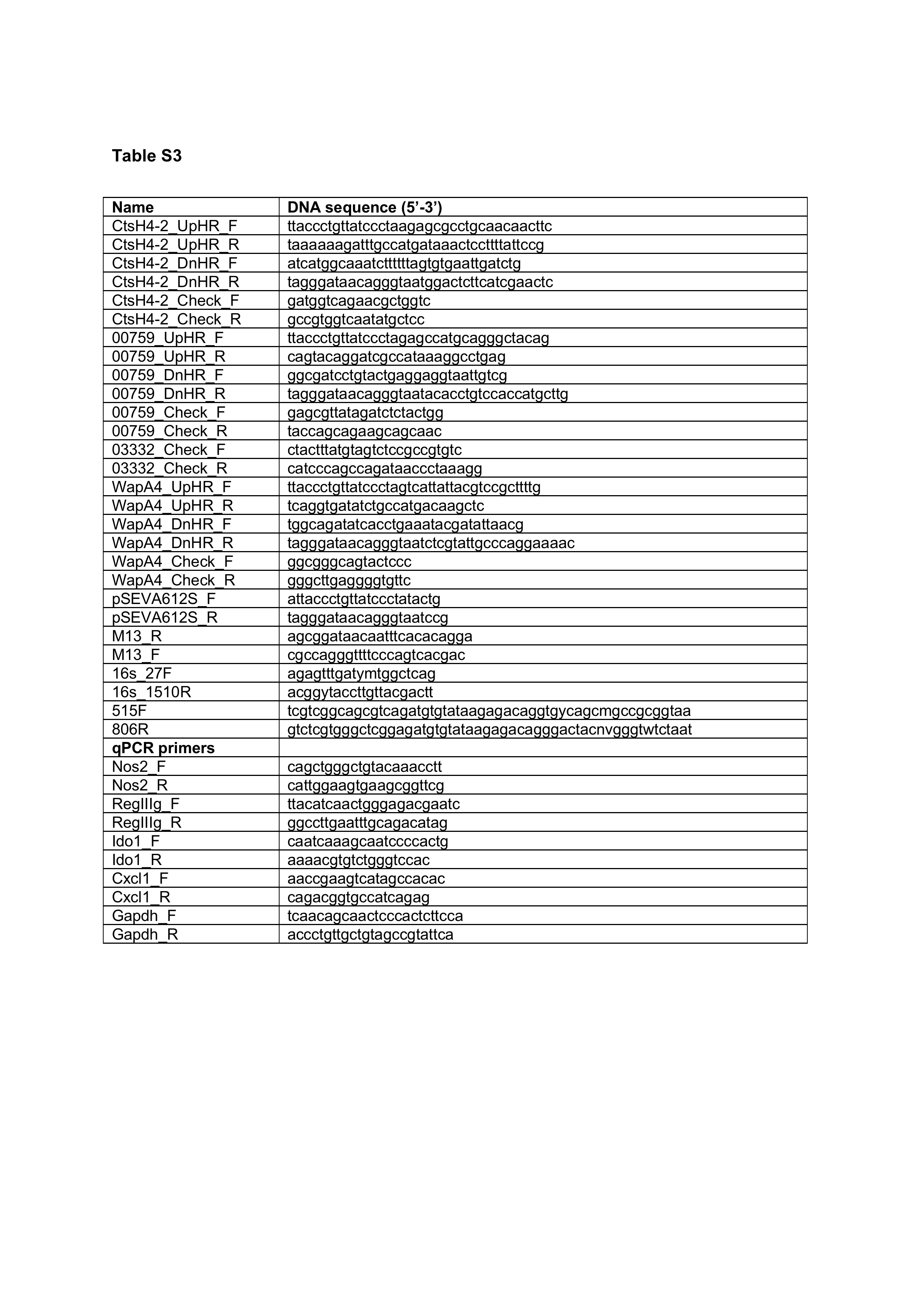

Supplement: TABLE S3 [file mbio.02410-21-st003.tif]
