# Supplementary material for: Citrobacter amalonaticus Inhibits the Growth of Citrobacter rodentium in the Gut Lumen
Source: mBio. 2021 Oct 5;12(5):e02410-21. doi: 10.1128/mBio.02410-21 (PMC8510533; doi:10.1128/mBio.02410-21)
Supplement: TABLE S2 [file mbio.02410-21-st002.pdf]

Table S2

Strain name	Description	Resistance	Reference
ICC169	*C. rodentium* WT, spontaneous Nal resistant mutant	Nal	(37)
ICC180	ICC169 constitutively expressing the *lux* bioluminescence cassette	Nal/Kan	(38)
ICC180 *ΔgrlR* (ICC1410)	ICC180 derivative, *ΔgrlR::CmR*	Nal/Kan/Cm	(14)
C. amalonaticus^{C3H} (ICC3000)	C3H/HeN non-permissive mouse stool isolate	N/a	This study
C. amalonaticus^{C3H} SmR (ICC3001)	Spontaneous streptomycin resistant mutant of *C. amalonaticus*^{C3H} (ICC3000)	Sm	This study
C. amalonaticus^{C3H} *ΔctsH4_2*	*C. amalonaticus*^{C3H}-SmR (ICC3001) lacking gene *ctsH4_2*	Sm	This study
C. amalonaticus^{C3H} *Δ03332*	*C. amalonaticus*^{C3H}-SmR (ICC3001) lacking gene *03332*	Sm	This study
C. amalonaticus^{C3H} *Δ00759*	*C. amalonaticus*^{C3H}-SmR (ICC3001) lacking gene *00759*	Sm	This study
C. amalonaticus^{C3H} *ΔwapA_4*	*C. amalonaticus*^{C3H}-SmR (ICC3001) lacking gene *wapA_4*	Sm	This study
Esherichia^{C57}	*Esherichia* genus isolate from uninfected C57BL/6 mouse stool	N/a	This study
Esherichia^{C57}-Rif	Spontaneous rifampicin resistant mutant of *Esherichia*^{C57}	Rif	This study
ICC169-GFP	ICC169 harbouring the pULTRA-GFP plasmid	Nal/Gm	This study
ICC169-RFP	ICC169 harbouring the pULTRA-RFP plasmid	Nal/Gm	This study
C. amalonaticus^{C3H}-*GFP*	*C. amalonaticus*^{C3H} SmR (ICC3001) harbouring the pULTRA-GFP plasmid	Sm/Gm	This study
C. amalonaticus^{C3H}-*RFP*	*C. amalonaticus*^{C3H} SmR (ICC3001) harbouring the pULTRA-RFP plasmid	Sm/Gm	This study
E. coli CC118λpir	Expressing the Pi protein for the replication of plasmids with the R6K origin	N/a	(39)
E. coli 1047 pRK2013	Helper strain for conjugation, Kanamycin-resistant.	Kan	(40)
