# Supplementary material for: Citrobacter amalonaticus Inhibits the Growth of Citrobacter rodentium in the Gut Lumen
Source: mBio. 2021 Oct 5;12(5):e02410-21. doi: 10.1128/mBio.02410-21 (PMC8510533; doi:10.1128/mBio.02410-21)
Supplement: TABLE S4 [file mbio.02410-21-st004.pdf]

Table S4

**Plasmid name**	**Description**	**Reference**
pSEVA612S	Integrative plasmid (ori R6K) that harbours the oriT for conjugation. Encoded Gm resistance.	(58)
pSEVA-ctsH4_2-HR	pSEVA612S derivative containing homology regions for the *C. amalonaticus*^{C3H} (ICC3001) ctsH4_2 gene	This study
pSEVA-00759-HR	pSEVA612S derivative containing homology regions for the *C. amalonaticus*^{C3H} (ICC3001) *00759* gene	This study
pSEVA-03332-HR	pSEVA612S derivative containing homology regions for the *C. amalonaticus*^{C3H} (ICC3001) *03332* gene	This study
pSEVA-wapA_4-HR	pSEVA612S derivative containing homology regions for the *C. amalonaticus*^{C3H} (ICC3001) *wapA_4* gene	This study
pACBSR	Expresses I-SceI and lambda-red induced by L-Arabinose. Encodes *Cm* resistance.	(59)
pULTRA-GFP	Replicating plasmid expressing GFP from the BioFab promoter	(60)
pULTRA-RFP	Replicating plasmid expressing RFP from the BioFab promoter	(60)
